# Structural brain correlates in major depressive disorder, anxiety disorders, and post-traumatic stress disorder: a diffusion tensor imaging meta-analysis

**DOI:** 10.1017/S0033291725100780

**Published:** 2025-07-21

**Authors:** Yunxiao Guo, Sijun Liu, Sara Poletti, Wei Liu, Yinong Liu, Junrong Zhao, Qian Xiong, Haixia Zheng, Dongtao Wei, Jiayi Li, Yuyi Zhang, Jun Ruan, Zhihong Ren

**Affiliations:** 1 Key Laboratory of Adolescent Cyberpsychology and Behavior (Ministry of Education); 2 Key Laboratory of Human Development and Mental Health of Hubei Province, National Intelligent Society Governance Experiment Base (Education), School of Psychology, Central China Normal University, Wuhan, China; 3 Psychiatry and Clinical Psychobiology, Division of Neuroscience, IRCCS Scientific Institute Ospedale San Raffaele, Milan, Italy; 4 Vita-Salute San Raffaele University, Milan, Italy; 5 Laureate Institute for Brain Research, Tulsa, OK, USA; 6 Oxley College of Health Sciences, The University of Tulsa, Tulsa, OK, USA; 7 Key Laboratory of Cognition and Personality of Ministry of Education, Faculty of Psychology, Southwest University, Chongqing, China; 8 School of Psychology, Liaoning Normal University, Dalian China

**Keywords:** anxiety disorders, comorbidities, diffusion tensor imaging, major depressive disorder, meta-analysis, post-traumatic stress disorder

## Abstract

The high comorbidity of major depressive disorder (MDD), anxiety disorders (ANX), and post-traumatic stress disorder (PTSD) complicates the study of their structural neural correlates, particularly in white matter (WM) alterations. Using fractional anisotropy (FA), this meta-analysis aimed to identify both unique and shared WM characteristics for these disorders by comparing them with healthy controls (HC). The aggregated sample size across studies includes 3,661 individuals diagnosed with MDD, ANX, or PTSD and 3,140 HC participants. The whole-brain analysis revealed significant FA reductions in the corpus callosum (CC) across MDD, ANX, and PTSD, suggesting a common neurostructural alteration underlying these disorders. Further pairwise comparisons highlighted disorder-specific differences: MDD patients showed reduced FA in the middle cerebellar peduncles and bilateral superior longitudinal fasciculus II relative to ANX patients and decreased FA in the CC extending to the left anterior thalamic projections (ATPs) when compared with PTSD. In contrast, PTSD patients exhibited reduced FA in the right ATPs compared to HC. No significant FA differences were observed between ANX and PTSD or between ANX and HC. These findings provide evidence for both shared and unique WM alterations in MDD, ANX, and PTSD, reflecting the neural underpinnings of the clinical characteristics that distinguish these disorders.

## Introduction

Major depressive disorder (MDD), anxiety disorders (ANX), and post-traumatic stress disorder (PTSD) are closely related disorders with both high degrees of comorbidity among them and shared risk factors (Ginzburg, Ein-Dor, & Solomon, 2010; Spinhoven et al., 2014). Besides this, they also display a range of distinctive epidemiological and clinical features. Depression and anxiety are common and often comorbid mental health disorders and their effects can broadly impact a person’s life. There is a plethora of evidence showing poorer quality of life, functional disability, and increased mortality burden in these patients (Han et al., 2021; Walker, McGee, & Druss, 2015).

In concrete terms, the phenotype of the three disorders is characterized by a high correlation between negative emotional states (Eysenck & Fajkowska, 2018), avoidance behaviors (Struijs et al., 2018), cognitive dysfunction, and rumination, resulting in psychological distress and impairments in daily functioning (Byllesby et al., 2016). Although the symptoms of PTSD are well outlined in the DSM-5-TR, the diagnostic specificity of PTSD still faces challenges due to the lack of objective biomarkers. The same is true for MDD and ANX, which are frequently comorbid with PTSD (Grupe & Nitschke, 2013; Rytwinski, Scur, Feeny, & Youngstrom, 2013). Most importantly, the heterogeneous nature of these disorders complicates the choice of effective treatments and hampers our ability to determine the efficacy of new treatments or to track temporal changes in the engagement of neural targets. This shared complexity across disorders underscores the need to better delineate white matter (WM) alterations that impact neural networks and cognitive and psychological function across these comorbid diagnoses and may help delineate subgroups of patients with similar characteristics.

While prior studies identified both shared and distinct gray matter alterations across MDD, ANX, and PTSD (Serra-Blasco et al., 2021), the WM microstructure remains underexplored. First, voxel-based morphometry studies have found lower gray matter volume (GMV) in the anterior cingulate (ACC) (Alves De Araujo Junior et al., 2023; Bora, Fornito, Pantelis, & Yücel, 2012; O’Doherty et al., 2015) and insular cortices (Sprengelmeyer et al., 2011; Yang et al., 2023). Second, diffusion tensor imaging (DTI) studies have found decreases in fractional anisotropy (FA) in the corpus callosum (CC) (Chen et al., 2017; Siehl et al., 2018; Zhou et al., 2022). However, besides sharing common features, each of the three disorders has specific and predominant symptom expressions, like sadness and coldness in MDD, worry and fear in ANX, and intrusive thoughts and avoidance in PTSD, as well as particular neural correlates. More importantly, although lower GMV in key regions such as ACC and insula and a decrease in FA in the CC have been suggested to be general markers of psychopathology (Kunimatsu et al., 2020), the literature is unclear on common and distinct WM characteristics of these disorders, as highlighted in the comparison between pediatric PTSD, MDD, and ANX (Kribakaran et al., 2020).

MDD, ANX, and PTSD exhibit distinct and overlapping features in clinical settings, particularly sharing neural mechanisms related to emotional processing, stress response, and cognitive control. Clinically, their high comorbidity rates and overlapping symptomatology, such as negative affect, avoidance, and dysregulation, have prompted increasing interest in transdiagnostic research approaches (Choi, Kim, & Jeon, 2020; Grisanzio et al., 2018; Post et al., 2021). In parallel, Neuroimaging studies reveal overlapping abnormalities in key regions, such as the amygdala and insula, which show hyperactivity across PTSD, social ANX, and specific phobia (Etkin & Wager, 2007). Altered neuropeptide Y levels further suggest common biological underpinnings in PTSD, MDD, and chronic stress (Tural & Iosifescu, 2020). The high comorbidity of PTSD and MDD may also reflect a trauma-related phenotype with shared neural impairments (Flory & Yehuda, 2015). WM, which supports connectivity within emotional regulation and cognitive control networks, plays a pivotal role in psychiatric disorders (Jiang et al., 2023; Zhang et al., 2018); therefore, examining its integrity may provide crucial insights into both shared and disorder-specific neural mechanisms (Mahon, Burdick, & Szeszko, 2010; Van Den Heuvel & Sporns, 2019). As a sensitive tool for assessing WM integrity, DTI offers valuable insights into these transdiagnostic mechanisms. Notably, scanner differences are a significant concern in neuroimaging studies (Jovicich et al., 2009; Nemoto et al., 2020), impacting image quality and measurement precision in DTI, particularly in terms of FA. Variations in magnetic field strength, gradient systems, and coil designs can affect resolution, signal-to-noise ratio, and diffusion sensitivity. This combination of sensitivity and diagnostic utility has solidified FA as a key metric in the field of neuroimaging research.

Emerging evidence suggests that MDD, ANX, and PTSD share alterations in key cognitive and emotional processing networks, yet each disorder also presents unique neurobiological characteristics (Brandl et al., 2022; Koopowitz, Zar, Stein, & Ipser, 2023; Serra-Blasco et al., 2021). Structural abnormalities in the CC have been reported across these disorders, indicating potential common disruptions in interhemispheric connectivity (Maxfield, McVilly, Devine, & Jordan, 2023; Yamada et al., 2015; Zhou et al., 2022). However, disorder-specific WM changes have also been noted, such as alterations in the anterior thalamic projection (ATP) in MDD (Guo et al., 2024; Henderson et al., 2013) and fronto-parietal and ventral attention networks in ANX (Baur, Hänggi, & Jäncke, 2012; Xu et al., 2019; Yang et al., 2019). Further, although PTSD was historically grouped within ANX, its distinct neural correlates warrant separate investigation. However, identifying disorder-specific structural alterations remains challenging, as structural magnetic resonance imaging studies often include participants with comorbidities (e.g., depression or other anxiety subtypes), making it difficult to isolate disorder-specific effects (Goodkind et al., 2015; Serra-Blasco et al., 2021). Moreover, reductions in FA in key regions, such as the CC and ATP, appear to be general markers of psychopathology. Therefore, conducting a meta-analysis with strict control of comorbidities is essential for examining disorder-specific WM alterations.

DTI provides a noninvasive method for observing microstructural alterations in WM. Over recent decades, it has been widely employed to investigate the directionality of WM tracts in the healthy human brain (Cattarinussi et al., 2022; Lin et al., 2024). FA, a key DTI metric, reflects the degree to which water diffusion is directionally constrained by WM microstructure. It captures critical properties, such as fiber density, axonal diameter, and myelination, offering a comprehensive index of WM integrity. Higher FA typically indicates more organized fiber architecture, whereas lower values suggest axonal or myelin disruption (Wallace, Mathias, & Ward, 2018). Compared to other DTI measures, such as mean or axial diffusivity, FA is considered a more sensitive marker for detecting microstructural abnormalities in WM (Gao et al., 2024; Zhao et al., 2021). Voxel-based analysis (VBA) is a whole-brain approach that assesses all WM voxels, including peripheral regions and fiber crossings. It reduces noise by identifying clusters of contiguous significant voxels (Nortje et al., 2013). In contrast, tract-based spatial statistics (TBSS) focuses on the central WM skeleton, highlighting clusters within regions with the highest FA values. While TBSS offers anatomical specificity, VBA enables voxel-wise analysis across the entire brain, potentially detecting broader WM abnormalities that TBSS might overlook (Di et al., 2018). This underscores the increasing need for comprehensive meta-analyses that integrate findings from TBSS and VBA research. Such analyses could help clarify the intrinsic WM alterations in MDD, ANX, and PTSD, highlighting both similarities and differences among these disorders and offering deeper insights into their pathogenesis.

Based on these findings, we hypothesized that reductions in FA would overlap across disorders in key cognitive and emotional brain networks, particularly in the CC. Additionally, we expected disorder-specific FA reductions, such as more prominent ATP abnormalities in MDD and FA differences in regions implicated in attentional and executive control networks in ANX. By synthesizing findings from different analytical methods and across multiple disorders, this meta-analysis aims to clarify both shared and unique WM alterations in MDD, ANX, and PTSD.

## Methods and materials

### Literature search

**Figure 1.**
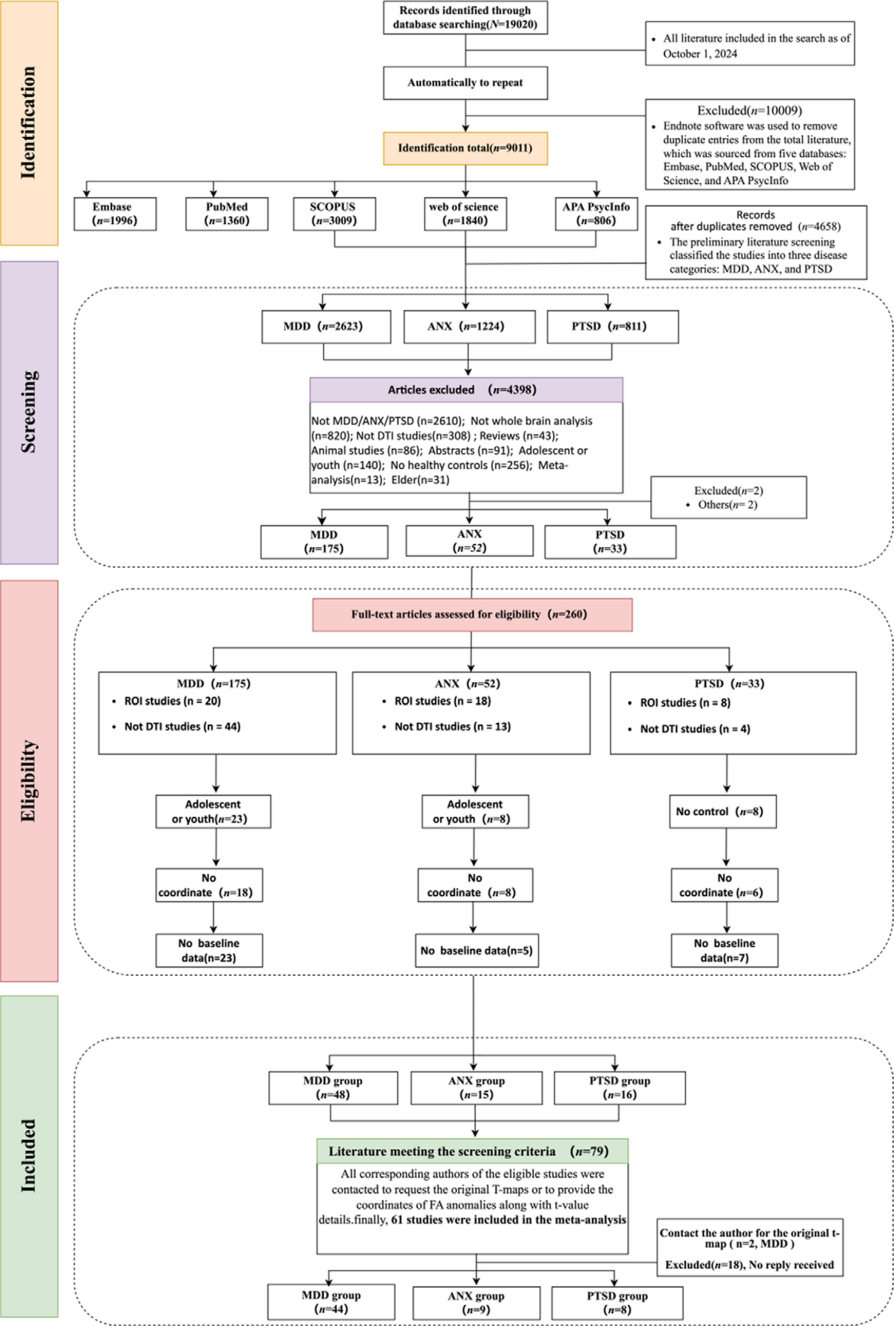
Flowchart of the systematic literature search. Footnote: ROI, ‘region of interests’; DTI, ‘diffusion tensor imaging’; MDD, ‘major depressive disorder’; ANX, ‘anxiety disorders’; PTSD, ‘post-traumatic stress disorder’.

Two independent reviewers (GYX. and LSJ) conducted an initial systematic literature search on November 6, 2024 (see Supplementary Appendix B). An extensive search of structural DTI studies comparing participants with MDD, ANX, and PTSD to healthy controls (HC) was performed across the Embase, Web of Science, PubMed, PsycINFO, Cochrane Library, and Scopus databases (Figure 1). Ultimately, among the 61 studies incorporated in our analysis, only one study within the PTSD meta-analysis accounted for comorbidity (among the 13 PTSD samples, there was one case each for current major depression, current panic disorder, and a history of panic disorder with agoraphobia) (Abe et al., 2006), with no other studies reporting any instances of comorbidity. The current meta-analysis followed the Preferred Reporting Items for Systematic Reviews and Meta-Analysis (PRISMA) guidelines. The protocol of this meta-analysis has been registered at PROSPERO (CRD42022374991).

### Quality assessment and data extraction

Four investigators (LYN, GYX, RJ, and LSJ) independently retrieved the relevant data from the studies. The data were then cross-checked by the same four investigators to minimize interpretation and data entry errors. The PRISMA guidelines, as recommended by Moher, Liberati, Tetzlaff, and Altman (2009), were followed throughout the process. The corresponding authors (or the principal investigators in case of no response) of all studies meeting the inclusion criteria were contacted via email and asked to provide the original T-maps and/or any missing relevant data. Two researchers responded, providing us with the original statistical T-maps, and were subsequently listed as co-authors of this paper (Poletti et al., 2018; Zheng et al., 2021). Additionally, the guidelines of Meta-analysis of Observational Studies in Epidemiology were followed in the present study (Stroup, 2000). The quality assessment of the included studies was performed using a 12-point checklist, as described in the Supplementary Quality Assessment. This checklist enabled the evaluation of multiple components, including the clinical and demographic characteristics of study samples, as well as the imaging-specific methodologies applied (Du et al., 2014). Two experienced authors (GYX and LSJ) independently assessed the methodological quality of the selected studies. Key data, such as coordinates and clinical details, were extracted independently and subsequently cross-verified. Any disagreements between the authors were resolved through discussion until a consensus was achieved.

### Differences in demographic variables

We calculated the mean and standard deviation for both age and age at onset, as well as the proportion of females in the samples. ANOVA and *χ* ^2^ tests were then performed to assess any demographic differences among patients with MDD, ANX, and PTSD. Statistical analyses were conducted using SPSS.

### Differences in WM FA

To identify alterations in FA among patients with MDD, ANX, PTSD, and HC, we performed a voxel-wise meta-analysis using the Seed-based d Mapping with Permutation of Subject Images (SDM-PSI) software (version 6.21, sdmproject.com) in accordance with standardized protocols. The complete SDM-PSI procedure is outlined in the tutorial available on the SDM Project website (Albajes-Eizagirre, Solanes, Vieta, & Radua, 2019) and further detailed in Supplementary Appendix C.

To enhance analytical sensitivity and in line with previous (Han et al., 2023; Zhao et al., 2022). We reported results using the default SDM kernel size and thresholds to optimally balance false positives and negatives (Fortier et al., 2024; Wang, Yan, & Lu, 2023). This approach applied an uncorrected *p*-value threshold of 0.005 and a minimum cluster size of 10 voxels, striking a balance between sensitivity and specificity by identifying meaningful effects while minimizing false positives (Müller et al., 2018; Radua et al., 2012). Additionally, we performed complementary analyses using threshold-free cluster enhancement (TFCE; 1000 permutations, *p* < 0.05, cluster extent ≥20 voxels) to correct for multiple comparisons. However, this conservative threshold may limit sensitivity, particularly in meta-analyses with a modest number of included studies (Radua et al., 2012). Therefore, we focused on the standard uncorrected threshold of *p* < 0.005, considering the relatively limited number of included studies, the imbalance in sample sizes across the three groups, and the likely subtle and potentially diffuse nature of WM changes in these disorders. We also assessed publication bias through visual inspection of the funnel plot and Egger’s test for a cautious interpretation of the results. Given the heterogeneity across disorders, we adopted a more lenient criterion (*p* < 0.05, uncorrected) for cluster sizes exceeding five voxels. Voxel-wise heterogeneity between studies was assessed using chi-square statistics, with a *χ* ^2^ *p*-value < 0.005, indicating significant heterogeneity (Wise et al., 2016). Publication bias was evaluated by extracting effect sizes and variances for each significant cluster peak from the effect size-signed differential maps of included studies. Funnel plots were visually inspected, and the Egger’s test was applied quantitatively. Symmetrical funnel plots and *p*-values (Egger’s test) ≥ 0.05 indicated an absence of publication bias. However, caution is advised when interpreting findings heavily influenced by a small number of studies or those with limited sample sizes.

### Meta-analyses

We conducted the following eight main meta-analyses: (1) all patients versus HC, (2) patients with MDD versus patients with ANX, (3) patients with MDD versus patients with PTSD, (4) patients with ANX versus patients with PTSD, (5) patients with MDD versus HC, (6) patients with ANX versus HC, and (7) patients with PTSD versus HC, and (8) patients with ANX and PTSD versus HC. We conducted all meta-analyses by incorporating the peak coordinates from all included studies or by using original t-maps. However, the number of included studies varied across disorders, with substantially more studies available for MDD than for ANX and PTSD. This imbalance may have influenced the statistical power and the ability to detect consistent WM alterations in ANX and PTSD. Given the limited number of studies on ANX and PTSD and the relatively larger volume of MDD research, our focus in the eight meta-analyses was primarily on identifying common abnormal brain regions when comparing MDD, ANX, and PTSD patients to HC, along with performing subgroup analyses.

To account for variability in study methodologies and clinical characteristics, as well as to assess the stability of shared WM abnormalities across the three disorders, we conducted a series of subgroup analyses comparing the MDD, ANX, PTSD, and HC groups. Subgroup analyses were first stratified by studies utilizing the VBA and TBSS methods. Second, analyses were performed based on studies applying corrected versus uncorrected statistical thresholds. Third, we compared first-episode with non-first-episode cases. Fourth, differences in WM abnormalities between medicated and unmedicated patients were examined. Meta-regressions were conducted to explore the influence of age and gender on neurobiological factors across all patients. Additionally, meta-regressions assessed the impact of psychotropic treatments both across all patients and within each mental disorder. For the MDD samples, meta-regressions further evaluated the effects of illness duration (in months) and depression severity on FA values. Depression severity was primarily assessed using the Hamilton Depression Rating Scale (HDRS-17), while studies employing Montgomery-Åsberg Depression Rating Scale (MADRS) scores were analyzed separately. we converted these to HDRS-17 equivalents using the formula: HDRS-17 = −1.58 + 0.86 × MADRS (33). We also explored potential publication bias for each cluster peak of statistically significant regions using funnel plots and Egger’s test.

## Results

### Included studies

The search retrieved 4398 studies potentially suitable (Figure 1), from which finally 61 met all the inclusion criteria, including participants being adults aged 18–65 years, performing whole-brain analysis, and providing a comparison between patients with MDD, ANX, or PTSD disorders and HC. Additionally, the studies had to provide a t-map or coordinates in Montreal Neurological Institute or Talairach space, with samples free of any comorbid neurological conditions and minimal current comorbid psychiatric disorders. A total of two original t-maps were achieved (MDD = 2) (Poletti et al., 2018; Zheng et al., 2021). However, 199 studies were excluded due to reasons such as adolescent or youth subjects, no coordinate, no baseline data, and other methodological issues. Among them, 131 studies were excluded from the MDD group, 43 studies were excluded from the ANX group, and 25 studies were discharged from the PTSD group. The exclusion of studies could introduce bias and limit the generalizability of the findings (See Table 1, 2, and 3 for more detailed information).

**Table 1.** the basic demographic characteristics of the MDD participants

Study	Method	Subjects, n (females, n)		Age, years		Time (month)	Severity M (SD)	Depression scale	Antidepressants(%)	comorbidities	Age at onset years (SD)	First-episode (Y/NA)	Age range
		Patients	Controls	MDD (M + SD)	HC (M + SD)								
Arnold et al., 2012	VBA	17 (13)	21 (14)	30.41 ± 11.35	26.90 ± 7.82	NA	9.18 (6.82)	HDRS 17-item	0	NA	NA	NA	21–51
Chen et al., 2022	TBSS	29 (24)	25 (23)	34.48 ± 14.83	35.00 ± 13.9	36.68 ± 30.81	22.79 ± 4.84	HDRS 17-item	0	NA	NA	Both	NA
Chhetry et al., 2016	VBA	16 (11)	12 (7)	34.40 ± 8.20	30.90 ± 7.90	NA	17.8 ± 3.85	HDRS–17-item	10	NA	NA	NA	22–50
De Diego-Adeliño et al., 2014	VBA	19 (11)	17 (12)	46.57 ± 7.87	43.40 ± 11.4	4.9 (2.88)	14.6 (7.5)	HAMD	100	NA	43.6 (6.8)	Y	NA
Dong et al., 2020	VBA	127 (69)	62 (37)	35.83 ± 9.17	35.01 ± 8.86	NA	31.48 ± 7.58	HAMD	0	NA	32.09 ± 9.11	Both	NA
Hayashi et al., 2014	TBSS	30 (13)	30 (13)	44.00 ± 12.00	44.00 ± 13.0	NA	20.6 ± 5.72	HAMD–17	0	NA	44 ± 12	Y	20–67
Jia et al., 2010	VBA	52 (27)	52 (28)	34.45 ± 13.10	37.10 ± 16.0	39.15 (59.72)	23.0077 (4.2527)	HAM-D score	0	NA	NA	NA	NA
Jiang et al., 2015	VBA	35 (18)	35 (17)	29.54 ± 8.57	31.91 ± 8.80	12.83 ± 17.02	27.70 ± 5.21	HDRS–17-item	0	NA	NA	Y	18–46
Jiang et al., 2021	VBA	82 (50)	72 (35)	34.00 ± 11.00	34.00 ± 12.0	6.5	23 (20–26)	HDRS	0	NA	NA	Y	18–45
Lai and Wu (2014)	TBSS	44 (23)	27 (15)	36.91 ± 5.31	38.29 ± 11.8	4.68 (1.44)	22.07 (2.29)	HAMD	0	NA	36.91 (5.31)	Y	NA
Liu et al., 2008	VBA	11 (5)	11 (5)	38.00 ± 10.00	37.00 ± 11.0	43.2 (76.8)	54 (13)	HAMD–24	0	NA	NA	Both	24–53
Liu et al., 2010	VBA	16 (12)	16 (12)	30.38	29.79	NA	22 (4.2)	HAMD–17	12.5	NA	NA	NA	21–35
Liu et al., 2016	TBSS	29 (12)	47 (10)	45.70 ± 12.50	41.80 ± 11.0	NA	21.4 (5.2)	HAMD–17	0	NA	45.7 (12.5)	Y	22–67
Lyon et al., 2019	TBSS	221 (115)	67 (34)	33.60 ± 11.70	30.30 ± 12.8	141.6 (127.2)	21.4 (3.7)	HDRS–17	NA	NA	21.8 (10.3)	NA	NA
Ma et al., 2007	VBA	14 (12)	14 (12)	28.90 ± 8.00	27.10 ± 6.7	10.3 (8.3)	37.5 (6.9)	BDI	0	NA	28.1 (7.8)	Y	20–41
Metin et al., 2020	TBSS	43 (21)	23 (9)	36.19 ± 12.75	30.52 ± 6.50	NA	NA	NA	NA	NA	NA	NA	14–62
Olvet et al., 2014	TBSS	52 (31)	46 (21)	35.25 ± 12.35	30.30 ± 9.30	74.33 (92.59)	19 (4.707)	HDRS–17-item	100	NA	NA	NA	18–65
Osoba et al., 2013	VBA	20 (8)	20 (3)	38.30 ± 11.60	33.80 ± 7.20	NA	15.1 (6.1)	HAMD	100	NA	NA	NA	NA
Repple et al., 2017	VBA	43 (24)	42 (21)	39.00 ± 11.70	36.10 ± 11.3	94.0 (118.0)	22.2 (5.0)	HAMD	100	NA	NA	NA	NA
Poletti et al., 2018	TBSS	65 (46)	59 (29)	49.49 ± 12.04	35.39 ± 13.0	NA	23.227 ± 5.647	HDRS–17-item	100	NA	35,15 ± 12,46	NA	NA
Seok et al., 2013	TBSS	86 (68)	62 (21)	44.70 ± 12.25	42.15 ± 14.5	42.67 (45.47)	14.6 ± 8.1	HDRS–17-item	52	NA	NA	NA	NA
Srivastava et al., 2016	VBA	15 (8)	15 (9)	30.14	29.13	10.4	19.47	HDRS–17-item	0	NA	NA	Y	18–45
Tatham et al., 2016	TBSS	55	18	36.40 ± 10.50	33.20 ± 10.2	NA	21.5 (3.95)	HDRS–17-item	0	NA	25.2 (10.6)	NA	19–58
Taylor et al., 2015	TBSS	74 (52)	91 (56)	35.50 ± 9.00	29.90 ± 9.10	NA	23.70 (4.43)	MADRS	0	NA	<35	NA	20–50
Tha et al., 2013	VBA	19 (7)	19 (6)	38.60 ± 13.5	36.50 ± 12.5	5.89 ± 5.69	19.00 ± 4.00	HDRS–17-item	0	NA	NA	NA	20–64
Walther et al., 2012	VBA	21 (11)	21 (9)	45.00 ± 13.70	41.00 ± 13.7	NA	26.4 (5.3)	HAMD	100	NA	NA	Both	NA
Wang et al., 2013	VBA	21 (16)	22 (14)	29.60 ± 12.60	30.20 ± 10.2	14.26 (13.4)	20.46 (6.3)	HDRS	0	NA	NA	NA	NA
Wu et al., 2011	VBA	23 (13)	21 (9)	31.40 ± 8.80	30.40 ± 8.20	2.17 ± 0.78	21.8 ± 3.8	HDRS	0	NA	31.4 ± 8.8	Y	18–45
Xiao et al., 2015	TBSS	22 (12)	22 (12)	20.14 ± 1.64	20.77 ± 1.41	NA	55.68 (5.75)	CESD	0	NA	20.14 ± 1.64	Y	18–24
Xu et al., 2021	VBA	45 (22)	41 (15)	33.16 ± 10.49	30.47 ± 8.08	NA	18.02 ± 4.79	HDRS–17-item	NA	NA	18.84 ± 9.49	NA	18–65
Yang et al., 2017	TBSS	30 (14)	28 (13)	29.33 ± 8.40	28.61 ± 6.92	6.20 ± 4.13	27.77 ± 4.73	HRSD–24-item	0	NA	29.33 ± 8.40	Y	NA
Zheng et al., 2021	TBSS	264 (186)	48 (22)	34.00 ± 10.66	33.30 ± 11.3	NA	61.79 ± 6.78	PROMIS	52	NA	NA	NA	18–55
Zhu et al., 2011	TBSS	25 (15)	25 (15)	20.55 ± 1.86	20.33 ± 1.68	10.34 ± 8.35	35.48 ± 6.70	CES-D	0	NA	20.23 (2.33)	Y	18–24
Zou et al., 2008	VBA	45 (30)	45 (30)	33.20 ± 8.90	31.00 ± 10.3	20.0 (18.4)	23.8 (SD 3.3)	HDRS	100	NA	NA	NA	18–55
Zuo et al., 2012	TBSS	16 (13)	19 (12)	37.00 ± 9.40	36.60 ± 7.70	NA	30.36 (6.2)	HAMD	0	NA	NA	NA	20–50
Guo et al., 2023	TBSS	33 (26)	34 (25)	25.61 ± 6.44	28.85 ± 7.94	1	23.27 (3.876)	HAMD	0	NA	25.61 ± 6.44	Y	NA
Vandeloo et al., 2023	TBSS	39 (17)	25 (10)	45.5 ± 14.4	43.0 (14.5)	51.69	34.4 (4.7)	MADRS	100	NA	NA	NA	18–65
Winter et al., 2023	TBSS	266 (207)	262 (194)	31.11 ± 11.37	31.40 ± 12.1	35.44 (59.42)	17.12 (11.24)	BDI	42	NA	22.78 ± 9.89	NA	18–65
Wu et al., 2023	TBSS	30 (17)	30 (18)	35.67 ± 7.86	36.53 ± 9.21	9.43 ± 1.97	NA	NA	100	NA	NA	NA	NA
Winter et al., 2023	TBSS	134 (105)	133 (98)	29.9 ± (10.8)	31.0 ± (11.7)	13	17.67 (10.94)	BDI	0	NA	NA	Y	18–65
F. F. Zhao et al., 2024	VBA	58 (31)	60 (31)	23	23.25	12	3.53 (1.13)	HAMD–17	0	NA	NA	Y	18–45
H. M. Zhao et al., 2024	TBSS	71 (59)	97 (64)	25.94 (6.31)	27.12 (7.48)	6.40 (3.96)	35.95 (8.10)	HAMD–24	0	NA	NA	Y	18–65
Flinkenflügel et al., 2023	TBSS	793 (515)	793 (502)	36.46 (13.17)	35.62 (12.96)	NA	9.17 (7.09)	HDRS–21	13	NA	NA	NA	18–65
Ma et al., 2023	TBSS	33 (17)	24 (10)	27.09 (8.94)	32.58 (12.10)	NA	20.79 (4.03)	HAMD–17	0	NA	NA	NA	20–65

Abbreviations: SD, standard deviation; VBA, voxel-based analysis; TBSS, tract-based spatial statistics; Time, Duration of onset; comorbidities, Whether there are other comorbidities; HAMD, Hamilton Rating Scale for Depression; CESD, Center for Epidemiologic Studies Depression Scale; HDRS, HAMD Rating Scale; MADRS, Montgomery-Asberg Depression Rating Scale; HDRS-17, the 17-item Hamilton Depression Rat; BDI, the Beck Depression Inventory Scale; PROMIS, PROMIS depression T-score; CBTM, cluster-based thresholding method; CES-D, catchment-area epidemiology survey depression; NA, no mentioned. Y, Yes N, No. Both: Both exist and the proportion is unclear.

**Table 2.** The basic demographic characteristics of the ANX participants

Study	Method	Subjects, n (females, n)		Age, years		Time of ANX (month)	Severity M (SD)	Measurement	Medicate (%)	Comorbidities	Age at onset years (SD)	First-episode(Y/NA)	Age range
		Patients	Controls	ANX (M + SD)	HC (M + SD)								
Baur et al., 2011	VBA	25 (7)	25 (7)	32 (10.4)	32 (10.1)	16 (10.6)	50 (11.2)	STAI	36	NA	15 ± 5. 9	NA	19–53
Kim et al., 2014	TBSS	36 (21)	27 (16)	38.83 (8.68)	38.15 (10.86)	50.24 (70.83)	PDSS:12.47 (5.77), BAI:25.8 (12.35)2.11 (3.4)	PDSS, BAI	100	NA	NA	NA	18–60
Kim et al., 2013	TBSS	26 (17)	26 (16)	37 (9.85)	37.62 (10.18)	NA	PDSS:11.1 (4.95), 10.86 (5.25) APPQ:42 (35.29), 44.43 (34.88) ASI-R:53.75 (24.41), 39.38 (18.89)	PDSS, APPQ, ASI-R	NA	NA	NA	NA	18–60
Lai and Wu (2013)	TBSS	30 (19)	21 (11)	47.03 (10.63)	41.14 (11.81)	NA	HARS:21.86 (2.71), 1.04 (0.97) PDSS:21.60 (2.26), 1.61 (1.20)	HARS, PDSS	NA	NA	NA	Y	NA
Lai and Wu (2016)	TBSS	53 (28)	54 (29)	43.283 (10.11)	40.38 (10.51)	5.35 (2.37)	HARS:23.35 (2.74), 1.25 (1.01) PDSS:21.43 (1.91) N/A	HARS, PDSS	NA	NA	NA	Y	NA
Lai et al., 2013	TBSS	21 (12)	21 (11)	46.61 (11.39)	41.14 (11.81)	4.8 (2.71)	HARS:22.42 (2.52) PDSS:22 (2)	HARS, PDSS	NA	NA	NA	Y	NA
Qiu et al., 2014	VBA	18 (6)	18 (6)	22.72 (3.85)	21.78 (3.9)	49.22 (40.17)	54.11 (11.9), 19.50 (8.5)	LSAS	NA	NA	NA	NA	N A
Wang et al., 2016	TBSS	28 (14)	28 (14)	32.93 (4.13)	33.21 (5.25)	29.75 (9.03)	GAD–7:1.96 (1.37), 16.82 (2.44) HAM-A:2.89 (2.04), 35.21 (6.53)	GAD–7, HAM-A	NA	NA	NA	Y	23–42
Zhang et al., 2013	VBA	16 (7)	26 (13)	30.38 (8.35)	30.62 (8.31)	11.56 (5.13)	25.94 (6.46)	HAM-A	NA	NA	NA	Y	NA

Abbreviations: SD, standard deviation; VBA, voxel-based analysis; TBSS, tract-based spatial statistics; time, duration of onset; comorbidities, whether there are other comorbidities; PDSS, Panic Disorder Severity Scale; BAI, Beck Anxiety Inventory; APPQ, Agoraphobic Cognitions Questionnaire; ASI-R, Anxiety Sensitivity Index-Revised; HARS, Hamilton Anxiety Rating Scale; GAD-7, Generalized Anxiety Disorder-7; HAM-A, Hamilton Anxiety Rating Scale; STAI, State–Trait Anxiety Inventory; LSAS, Liebowitz Social Anxiety Scale; NA, No mentioned. Y, Yes N, No.

**Table 3.** The basic demographic characteristics of the PTSD participants

Study	Method	Subjects, n (females, n)		Age, years		Time of PTSD (month)	Severity M (SD)	Measurement	Medicate (%)	Comorbidities	Age at onset years (SD)	First-episode(Y/NA)	Age range
		Patients	Controls	PTSD (M + SD)	HC (M + SD)								
Chen et al., 2021	VBA	27 (20)	30 (23)	48.4 (10.3)	49.9 (6.1)	NA	78.2 (19.3)	CAPS	0	NA	NA	NA	18–65
Odoherty et al., 2018	VBA	25 (13)	25 (13)	34 (8.4)	31.7 (6)	NA	95.6 (25.9)	CAPS	NA	NA	NA	NA	18–50
Abe et al., 2006	VBA	9 (4)	16 (6)	44.6 (16)	44.4 (14)	NA	62.2 (18)	CAPS	NA	current major depression (n = 1), current panic disorder (n = 1) and a history of panic disorder with agoraphobia (n = 1)	NA	NA	24–69
Xi et al., 2013	TBSS	17 (0)	17 (0)	31 (7)	32 (6)	NA	NA	NA	0	NA	NA	NA	20–45
L. Li et al., 2016	VBA	88 (60)	91 (66)	43 (10.4)	43.7 (9.9)	NA	62.9 (10.6)	CAPS	0	NA	NA	Y	NA
Sun et al., 2013	VBA	21 (13)	22 (13)	40.86 (12.26)	40.23 (12.54)	NA	44 (14.92)	CAPS	0	NA	NA	NA	18–60
Zhang et al., 2012	VBA	13 (0)	14 (0)	37.54 (3.69)	40.86 (5.2)	NA	55.07 (12.95)	PCL-C	NA	NA	NA	NA	NA
Fani et al., 2012	VBA	25 (25)	26 (26)	34 (12.5)	38 (12.8)	NA	23.3 (8.8)	PSS	0	NA	NA	NA	20–62

Abbreviations: SD, standard deviation; VBA, voxel-based analysis; TBSS, tract-based spatial statistics; time, duration of onset; comorbidities, whether there are other comorbidities. CAPS, Clinician-Administered PTSD Scale; PCL-C, PTSD Checklist-Civilian Version; NA, No mentioned. Y, Yes.

### Sample characteristics

The MDD meta-analysis included 44 studies. The number of MDD patients is 3,183, and the total number of HCs is 2,653. When Magnetic Resonance Imaging (MRI) assessments were conducted, 664 patients were classified as first-episode cases (excluding ‘both’ in the table, with only ‘Y’ totaling 664 out of 852). The average HDRS-17 score for these first-episode patients was 25.48 (excluding ‘both,’ which averaged 25.48, while ‘both’ averaged 24.33, although ‘both’ only had one entry, whereas ‘Y’ had two). In studies marked as ‘both,’ indicating a mix of first-episode and non-first-episode patients, the specific proportions of each group were unclear.

A total of nine studies were included in the ANX meta-analysis. The total number of ANX patients is 253 and the number of HC patients is 246. Of the 253 patient participants, 166 (65.61%) were diagnosed with panic disorder, 43 (16.99%) with social ANX, and 44 (17.39%) with generalized ANX.

The total number of PTSD patients is 225, compared with 241 HC. Among the studies reporting on medication use, 45 patients (17.78%) were taking antidepressants or anti-anxiety medications. PTSD was included in eight studies with 225 participants, and the comorbidity rate of PTSD with other disorders was 1.3%.

### Age and gender differences

See Supplementary Appendix D.

### Regional differences in WM

#### Conjunction analysis (MDD, ANX, and PTSD vs. HC)

**Figure 2.**
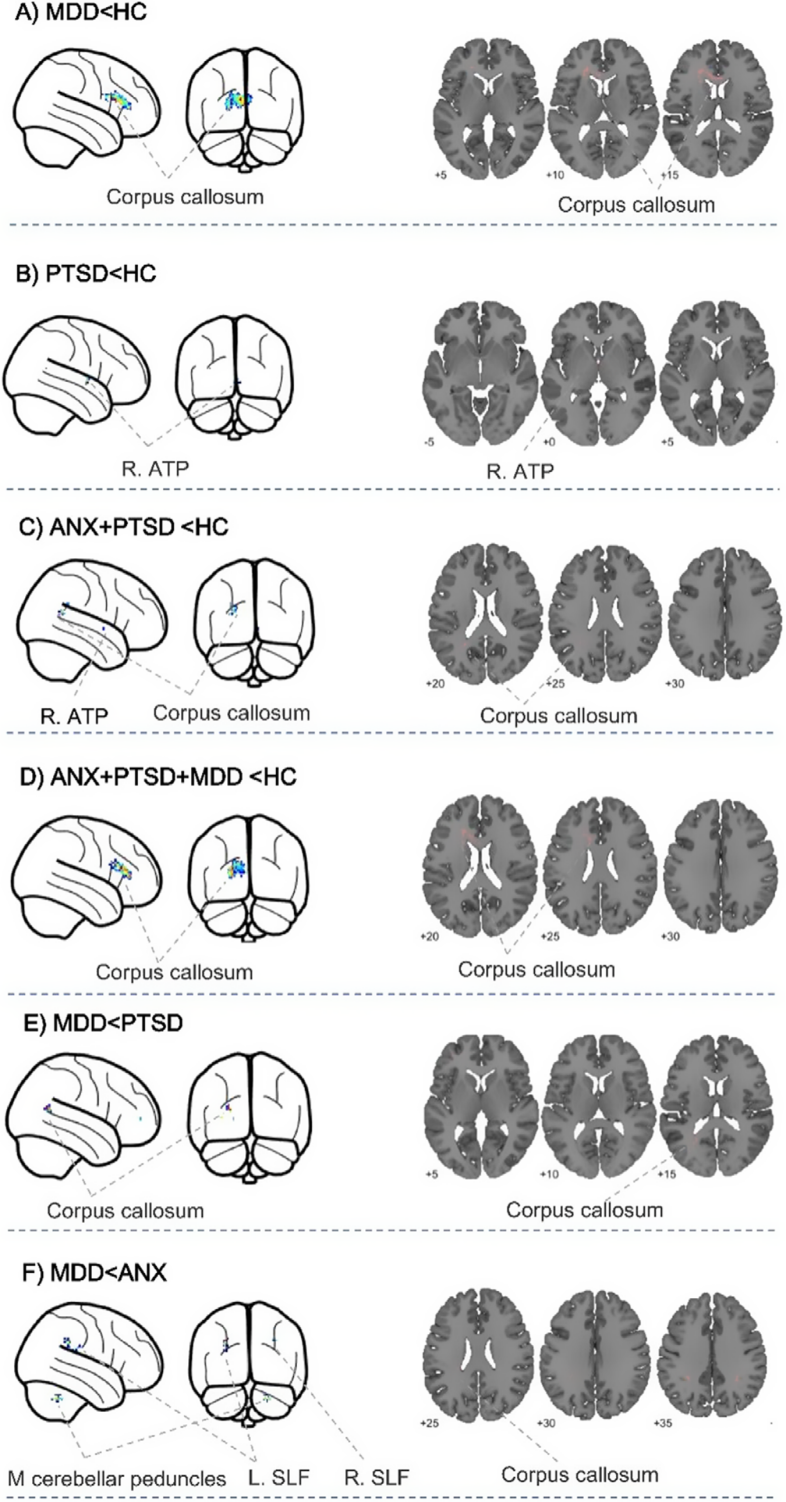
The results of regional differences in white matter. MDD, ‘major depressive disorder’; ANX, ‘anxiety disorders”; PTSD, ‘post-traumatic stress disorder’; ATP, ‘anterior thalamic projection’; SLF, ‘superior longitudinal fasciculus’; L, ‘Left’; R, ‘Right’.

Among the 61 studies included in the meta-analysis, the pooled meta-analysis revealed significant FA reductions in the CC in MDD, ANX, and PTSD compared with HC (*p* < 0.005, uncorrected) (see Table 2 in Supplementary Appendix G and Figure 2).

#### MDD versus ANX analysis

In comparison to findings from nine ANX studies, data from 44 MDD studies indicated that MDD patients exhibited reduced FA in the middle cerebellar peduncles and bilateral superior longitudinal fasciculus II (*p* < 0.05, uncorrected) (see Table 4 in Supplementary Appendix G and Figure 2).

#### MDD versus PTSD analysis

Compared to findings from eight PTSD studies, data from 44 MDD studies demonstrated reduced FA in MDD patients, specifically in the CC extending to the left ATP (*p* < 0.05, uncorrected) (see Table 5 in Supplementary Appendix G and Figure 2).

#### ANX versus PTSD analysis

No significant differences in FA were observed in ANX (nine studies) patients compared to PTSD (eight studies) patients at any threshold.

#### MDD versus HC analysis

In MDD (44 studies) patients, FA was reduced in the CC extending to the left ATP when compared to HC (*p* < 0.05, uncorrected) (see Table 1 in Supplementary Appendix G and Figure 2).

#### ANX versus HC analysis

No significant differences in FA were observed in ANX (9 studies) patients compared to HC at any threshold.

#### PTSD versus HC analysis

In contrast, PTSD (eight studies) patients showed lower FA in the right ATP relative to HC (*p* < 0.05, uncorrected) (see Table 3 in Supplementary Appendix G and Figure 2).

#### Conjunction analysis (ANX, PTSD vs. HC)

Across 9 ANX studies and 44 MDD studies compared to HC, reduced FA was identified in the CC extending to the left ATP (*p* < 0.05, uncorrected) (see Table 6 in Supplementary Appendix G and Figure 2).

#### All participants subgroup analysis (VBA, TBSS, NO-medicine)

**Figure 3.**
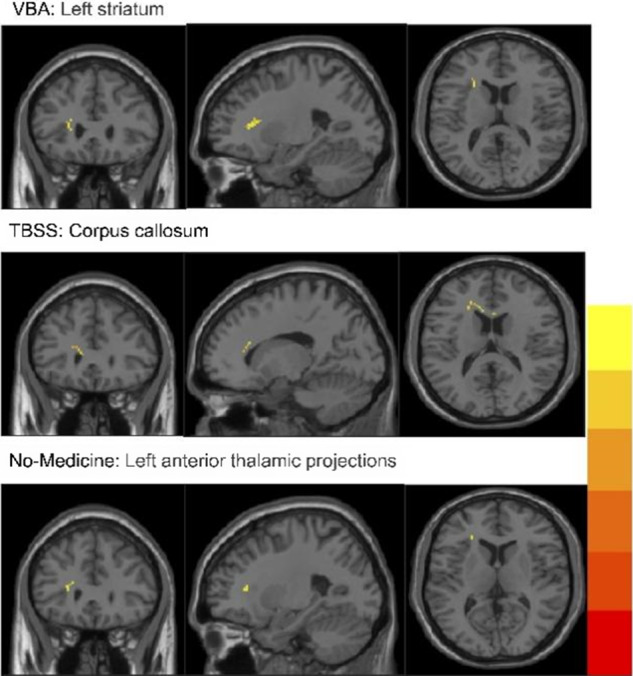
Results of subgroup analysis of regional differences in white matter. VBA, ‘Voxel-based analysis’; TBSS, ‘tract-based spatial statistics’.

Subgroup analysis of all participants revealed a significant reduction in FA within the left striatum for all three disorders compared to HC in the VBA results (*p* < 0.05, uncorrected) (see Table 9 in Supplementary Appendix F). The TBSS results further indicated a significant FA reduction in the CC across all three disorders (*p* < 0.05, uncorrected). As detailed in Table 8 of Supplementary Appendix G, for untreated patients, FA was also significantly reduced in the left ATP within the CC across the three disorders (*p* < 0.05, uncorrected) (see Table 7 in Supplementary Appendix F). All the subgroup analysis results of regional differences in WM are shown in Figure 3.

### Meta-regressions

Meta-regression analyses indicated no linear associations between the mean age of patients, percentage of female patients, or depression duration and FA abnormalities in MDD. Additionally, due to limited data on ANX and PTSD in the studies, no meta-regression analyses were conducted for these disorders.

### Publication bias and heterogeneity tests

All results from the meta-analyses and meta-regressions were nonsignificant in Egger’s tests (*p* > 0.05), indicating no evidence of publication bias. Furthermore, *I* ^2^ values ranged between 0 and 40%, implying low to negligible heterogeneity issues (Higgins & Cochrane Collaboration, 2020).

## Discussion

This meta-analysis is the first to quantitatively assess WM alterations across MDD, ANX, and PTSD using both reported coordinates and original t-maps. Our findings reveal reduced FA in the CC in MDD patients compared to HC, while PTSD patients showed reduced FA in the right ATP. No significant FA changes were observed in ANX. The joint analysis identified the CC as a commonly affected WM region across all three disorders, with disorder-specific patterns of abnormalities. These results build on prior research, offering a more integrated understanding of WM alterations. Given the limited studies on PTSD and ANX, we applied a more lenient threshold to explore abnormalities, while for other analyses, we used an uncorrected p-value of 0.05 as a more liberal approach to detect potential differences, acknowledging that some observed effects involve tiny clusters, which should be interpreted with caution (Radua et al., 2012).

However, it is important to note that while structural changes in the CC were commonly observed across MDD, ANX, and PTSD, the underlying neurobiological mechanisms for these alterations remain speculative. Potential contributing factors include changes in axonal transport, myelination, or myelin loss, and these may vary across the disorders studied. Additionally, the overlap in WM changes across the three disorders suggests a shared vulnerability in specific neural pathways, although the precise mechanisms remain to be elucidated. These findings support previous research while offering novel insights into the relationship between WM abnormalities and these psychiatric disorders.

The CC’s involvement in each disorder highlights its importance as a shared region of interest. Specifically, the MDD group exhibited reduced FA in the CC, whereas the PTSD group showed alterations in the ATP. These disorder-specific patterns suggest that, while there is a commonality in WM abnormalities, the underlying pathological mechanisms may differ between these disorders. Additionally, it is crucial to recognize that the course of the illness may impact the extent of structural changes, with longer disease duration potentially exacerbating WM alterations (Zhao et al., 2021).

### Conjunction analysis

The pooled meta-analysis revealed significant FA reductions in the CC among ANX, PTSD, and MDD patients compared to HC, supporting our hypothesis of shared WM abnormalities across these disorders. This finding contrasts with the gray matter results by Serra-Blasco et al. (2021), who reported no significant structural overlap, suggesting distinct patterns in gray and WM changes. The CC, the brain’s largest myelinated tract with over 300 million fibers, plays a critical role in integrating motor, perceptual, cognitive, and emotional functions between hemispheres. Disruptions in the CC may impair interhemispheric communication, contributing to deficits in emotional regulation, memory, and cognition (Sridharan, Levitin, & Menon, 2008; Tham et al., 2011; van Velzen et al., 2020). CC plays a crucial role in regulating cognitive processes and facilitating communication between the two hemispheres of the brain (Yamada et al., 2015). The results of this study suggest that abnormalities in the CC may represent a common mechanism across all three disorders.

In terms of psychological mechanisms and clinical manifestations, the phenotypes of MDD, ANX, and PTSD are characterized by prominent negative emotional states (Eysenck & Fajkowska, 2018). They also exhibit significant avoidance behaviors, which contribute to the development and maintenance of ANX and PTSD and may further mediate the progression to MDD (Struijs et al., 2018). Neurobiologically, these disorders share a complex hereditary predisposition involving genes related to monoamine systems, neuropeptides, and the hypothalamic–pituitary–adrenal axis, which collectively influence normal stress response mechanisms (Smoller, 2016).

This study is the first to identify a shared brain region with WM FA abnormalities across patients with MDD, ANX, and PTSD, potentially linking this common abnormality to shared clinical features. These findings offer valuable insights into the neural mechanisms underlying these disorders. The CC, highly susceptible to environmental stressors, may exhibit FA alterations due to of stress, a common factor in MDD, ANX, and PTSD. Given the CC’s critical role in memory and cognition, these alterations could underlie the cognitive impairments observed in these disorders.

The subgroup analysis utilized VBA and TBSS methods to investigate the common WM regions affected across the three disorders; however, the results contradicted the initial hypothesis. Specifically, VBA identified significant abnormalities not in the CC, as expected, but rather in the striatum. In contrast, the TBSS analysis reaffirmed the CC findings, providing consistent results. These findings may suggest that abnormal neural circuits involving both the CC and the striatum represent a distinctive shared mechanism in the WM of individuals with these three disorders. The neurobiological mechanisms underlying mental illness remain complex and not yet fully understood. Previous studies have identified extreme regional heterogeneity in GMV deviations as a common feature of various mental illnesses (Segal et al., 2023). These deviations are frequently associated with functional circuits and networks, indicating that neural circuits formed by connections between multiple brain regions may serve as more reliable markers of mental illness. WM, as a network of nerve fibers connecting regions of gray matter, plays a critical role in supporting these circuits (Ribeiro et al., 2024). Although the subgroup analysis results offer new insights, caution is warranted in their interpretation due to the imbalance in the number of references between ANX, PTSD, and MDD.

### MDD meta-analyses

The results of the MDD meta-analyses consolidate previously identified WM alterations, underscoring the significance of CC abnormalities in MDD. Thus, we propose that CC abnormalities may play a crucial role in the pathophysiology of MDD. Our findings align with recent meta-analyses on MDD, further emphasizing the central role of the CC in this condition (Chen et al., 2017; Guo et al., 2024; Jiang et al., 2017; Zhou et al., 2022).

In terms of MDD neurological function, the genu connects the prefrontal and orbitofrontal regions, and the body connects the precentral frontal regions and parietal lobes (Liao et al., 2014). Research suggests that abnormalities in the anterior genu of the CC may impair interhemispheric connectivity for MDD patients (Xu et al., 2013). Reduced FA in the CC could indicate an impairment in the transfer of information between the hemispheres of the frontal lobe, which may lead to working memory and emotional processing issues in patients with MDD (Aghajani et al., 2014). Older patients with MDD demonstrated alterations in the WM integrity of the CC (Han et al., 2014). Our study confirmed the presence of WM abnormalities in the CC in adults with depression, suggesting that these disruptions may be fundamental to MDD pathophysiology and persist across an individual’s lifespan. These findings add to the evidence that compromised WM integrity within the CC may represent an intrinsic neuropathological feature linked to the development of MDD. Our meta-analysis found reduced FA in the left ATP, which connects the thalamus to the prefrontal cortex via the anterior limb of the internal capsule. The prefrontal cortex is responsible for executive function and complex behavior planning (Zhou et al., 2022). ATP abnormalities may be associated with behavioral abnormalities in MDD patients (Jiang et al., 2017). Previous neuroimaging studies found that depression severity correlated with decreased WM integrity in ATP (Henderson et al., 2013). A study found that young adults and adolescents experiencing depression had abnormal ATP functions (Bessette et al., 2014).

### ANX meta-analysis

Under conventional thresholds used in previous studies, no significant differences in FA were observed between ANX patients and HCs. However, when a more lenient threshold was applied, the meta-analysis revealed significant FA reductions in the right inferior network and inferior fronto-occipital fasciculus in ANX patients compared to HCs (*p* < 0.1, uncorrected). Although these findings should be interpreted with caution (see Supplementary Appendix E), they provide a preliminary foundation for future research in this area.

### PTSD meta-analysis

The meta-analysis found FA reductions in the right ATP and CC in patients with PTSD compared to those in HC. This result is consistent with the MDD meta-analysis. It suggests that there may be a common neural mechanism in the WM of the brain for these two diseases. Previous studies have found CC abnormalities in PTSD patients compared with HC (Graziano, Bruce, & Paul, 2021). In a comprehensive study recently, researchers compared the regional WM metrics of 1426 individuals with PTSD and 1621 controls (consisting of 2174 males and 873 females aged between 18 and 83) (Dennis et al., 2021). The findings revealed that individuals with PTSD exhibited disrupted WM organization, particularly in the tapetum region of the CC, which was indicated by lower FA. The commissural and association fibers, thalamic radiations, left superior longitudinal fasciculus, and superior and forceps major showed FA reductions associated with depression in another large sample study of the UK Biobank sample (Shen et al., 2017). Consistent with our meta-analysis, these results suggest that there might be a shared brain mechanism among individuals with PTSD and depression.

Besides, prethalamic radiation transmits information from the hippocampus to the ACC gyrus through the thalamus (Mamah et al., 2010). The hippocampus files new memories for long-term declarative memory. The ACC recognizes and evaluates emotional information while regulating responses. Previous functional MRI (fMRI) studies have shown significant activation of the ACC in PTSD patients during trauma-related image stimulation, suggesting its involvement in the emotional processing of traumatic information (Frewen et al., 2008; Lanius et al., 2007). The reduced FA in the right anterior thalamic radiation observed in PTSD patients may reflect disruptions associated with traumatic memories, aligning with clinical symptoms such as memory loss. This meta-analysis highlights structural abnormalities in the medial prefrontal lobe, as evidenced by decreased FA in the right ATP, and reinforces prior findings on disrupted anatomical connectivity in PTSD. Although the exploratory analysis for ANX and PTSD aligns with previous research, these findings should be interpreted cautiously, given the use of lenient thresholds.

Additionally, the exclusion of a large number of studies that did not meet the inclusion criteria may affect the comprehensiveness of the analysis. While the majority of studies included in this meta-analysis focused on MDD, there were fewer studies available on anxiety and PTSD. This imbalance in the number of studies could impact the overall conclusions and should be considered when interpreting the results.

### Limitations

Several limitations should be acknowledged. First, despite contacting all authors for raw statistical maps, only two provided them, limiting the dataset and statistical power. Future studies with larger collections of raw statistical maps are needed. Second, this study focused exclusively on non-elderly adult populations due to the scarcity of whole-brain DTI studies in adolescents and older adults, preventing comparisons of FA differences across age groups. Third, variability in data acquisition and processing, such as MRI field strength, voxel resolution, diffusion directions, slice thickness, and software, may have influenced the precision of our findings. Scanner differences, small sample sizes, and lack of scanner-specific parameters compounded this issue. Fourth, clinical factors such as cognitive function and psychological status, were not fully controlled, potentially introducing confounders, while insufficient reporting limited subgroup and meta-regression analyses. Fifth, although we aimed to include all relevant whole-brain DTI studies, publication bias cannot be entirely ruled out, even for studies with null results. Fifth, the results of this meta-analysis should be interpreted considering scanner differences, and variations in scanner hardware, such as diffusion sensitivity, could still impact image quality and FA measurements (Jovicich et al., 2009; Nemoto et al., 2020). Finally, given the exploratory nature of our study, we applied a lenient uncorrected threshold (*p* < 0.005) to enhance sensitivity. Although this approach balances false positives and negatives, the results should be interpreted cautiously, and future studies are encouraged to adopt more stringent corrections, such as TFCE, to validate these findings. The imbalance in study numbers, with fewer studies for ANX and PTSD compared to MDD, may have further reduced the statistical power and influenced the generalizability of our conclusions, particularly regarding treatment efficacy across these conditions. Although the exclusion of studies from our analysis was based on specific criteria to ensure the reliability of our findings, this exclusion could limit the scope of the meta-analysis. To enhance the robustness of findings, future research should aim to include a more balanced representation of studies across these disorders and seek validation using independent datasets. While our meta-analysis provides valuable insights into the WM abnormalities shared by MDD, ANX, and PTSD, we recognize that further validation in larger, more diverse clinical populations, and the integration of additional neurobiological markers, will be necessary to strengthen the clinical relevance of these findings. These steps will be critical for advancing their application to clinical practice in the future.

## Conclusion

This meta-analysis highlights both shared and disorder-specific WM alterations in MDD, ANX, and PTSD, with significant reductions in the CC observed across all three conditions, suggesting common neurobiological pathways underlying their high comorbidity. Disorder-specific differences were identified, such as reduced FA in the middle cerebellar peduncles and superior longitudinal fasciculus II in MDD compared to ANX and distinct alterations in ATP in PTSD. These findings underscore the role of structural WM differences in mediating unique clinical features while pointing to overlapping neural mechanisms across these disorders, advancing our understanding of their neurobiological underpinnings.

## Supporting information

Guo et al. supplementary material Guo et al. supplementary material

## Supplementary material

The supplementary material for this article can be found at http://doi.org/10.1017/S0033291725100780.
