# Supplementary material for: Structural brain correlates in major depressive disorder, anxiety disorders, and post-traumatic stress disorder: a diffusion tensor imaging meta-analysis
Source: Psychol Med. 2025 Jul 21;55:e203. doi: 10.1017/S0033291725100780 (PMC12315647; doi:10.1017/S0033291725100780)
Supplement: Guo et al. supplementary material [file S0033291725100780sup001.docx]

**Structural brain correlates in major depressive disorder, anxiety disorders, and post-traumatic stress disorder: A diffusion tensor imaging meta-analysis studies**

**Online Supplementary Material**

**Appendix A. PRISMA checklist**

**Appendix B. Search Strategy**

**Appendix C Method of SDM-PSI**

**Appendix D Age and gender differences**

**Appendix E: Anxiety disorders meta-analysis**

**Appendix F. Risk of Bias**

**Appendix G.** **Coordinates for each result**

**Appendix H.** **DTI analysis methods and statistical characteristics of the participants**

| **Section and Topic** | **Item #** | **Checklist item** | **Location where item is reported** |
| --- | --- | --- | --- |
| **TITLE** | | | 1 |
| Title | 1 | Identify the report as a systematic review. | 1 |
| **ABSTRACT** | | | 1 |
| Abstract | 2 | See the PRISMA 2020 for Abstracts checklist. | 1 |
| **INTRODUCTION** | | | 1-4 |
| Rationale | 3 | Describe the rationale for the review in the context of existing knowledge. | 1-3 |
| Objectives | 4 | Provide an explicit statement of the objective(s) or question(s) the review addresses. | 3-4 |
| **METHODS** | | | 4-7 |
| Eligibility criteria | 5 | Specify the inclusion and exclusion criteria for the review and how studies were grouped for the syntheses. | 4 |
| Information sources | 6 | Specify all databases, registers, websites, organisations, reference lists and other sources searched or consulted to identify studies. Specify the date when each source was last searched or consulted. | 4 |
| Search strategy | 7 | Present the full search strategies for all databases, registers and websites, including any filters and limits used. | 4 |
| Selection process | 8 | Specify the methods used to decide whether a study met the inclusion criteria of the review, including how many reviewers screened each record and each report retrieved, whether they worked independently, and if applicable, details of automation tools used in the process. | 4-5 |
| Data collection process | 9 | Specify the methods used to collect data from reports, including how many reviewers collected data from each report, whether they worked independently, any processes for obtaining or confirming data from study investigators, and if applicable, details of automation tools used in the process. | 4-5 |
| Data items | 10a | List and define all outcomes for which data were sought. Specify whether all results that were compatible with each outcome domain in each study were sought (e.g. for all measures, time points, analyses), and if not, the methods used to decide which results to collect. | 4-5 |
|  | 10b | List and define all other variables for which data were sought (e.g. participant and intervention characteristics, funding sources). Describe any assumptions made about any missing or unclear information. | 4-5 |
| Study risk of bias assessment | 11 | Specify the methods used to assess risk of bias in the included studies, including details of the tool(s) used, how many reviewers assessed each study and whether they worked independently, and if applicable, details of automation tools used in the process. | 4-5 |
| Effect measures | 12 | Specify for each outcome the effect measure(s) (e.g. risk ratio, mean difference) used in the synthesis or presentation of results. | 5-7 |
| Synthesis methods | 13a | Describe the processes used to decide which studies were eligible for each synthesis (e.g. tabulating the study intervention characteristics and comparing against the planned groups for each synthesis (item #5)). | 7 |
|  | 13b | Describe any methods required to prepare the data for presentation or synthesis, such as handling of missing summary statistics, or data conversions. | 6 |
|  | 13c | Describe any methods used to tabulate or visually display results of individual studies and syntheses. | 6 |
|  | 13d | Describe any methods used to synthesize results and provide a rationale for the choice(s). If meta-analysis was performed, describe the model(s), method(s) to identify the presence and extent of statistical heterogeneity, and software package(s) used. | 5-7 |
|  | 13e | Describe any methods used to explore possible causes of heterogeneity among study results (e.g. subgroup analysis, meta-regression). | 5-7 |
|  | 13f | Describe any sensitivity analyses conducted to assess robustness of the synthesized results. | 5-7 |
| Reporting bias assessment | 14 | Describe any methods used to assess risk of bias due to missing results in a synthesis (arising from reporting biases). | 6-7 |
| Certainty assessment | 15 | Describe any methods used to assess certainty (or confidence) in the body of evidence for an outcome. | 6 |
| **RESULTS** | | | 8-11 |
| Study selection | 16a | Describe the results of the search and selection process, from the number of records identified in the search to the number of studies included in the review, ideally using a flow diagram. | 29 |
|  | 16b | Cite studies that might appear to meet the inclusion criteria, but which were excluded, and explain why they were excluded. | 4 |
| Study characteristics | 17 | Cite each included study and present its characteristics. | 31 |
| Risk of bias in studies | 18 | Present assessments of risk of bias for each included study. | Appendix C. Risk of Bias |
| Results of individual studies | 19 | For all outcomes, present, for each study: (a) summary statistics for each group (where appropriate) and (b) an effect estimate and its precision (e.g. confidence/credible interval), ideally using structured tables or plots. | Appendix D. Coordinates for each result |
| Results of syntheses | 20a | For each synthesis, briefly summarise the characteristics and risk of bias among contributing studies. | 8 |
|  | 20b | Present results of all statistical syntheses conducted. If meta-analysis was done, present for each the summary estimate and its precision (e.g. confidence/credible interval) and measures of statistical heterogeneity. If comparing groups, describe the direction of the effect. | 8-11 |
|  | 20c | Present results of all investigations of possible causes of heterogeneity among study results. | 11 |
|  | 20d | Present results of all sensitivity analyses conducted to assess the robustness of the synthesized results. | 11 |
| Reporting biases | 21 | Present assessments of risk of bias due to missing results (arising from reporting biases) for each synthesis assessed. | 11 |
| Certainty of evidence | 22 | Present assessments of certainty (or confidence) in the body of evidence for each outcome assessed. | 11 |
| **DISCUSSION** | | | 11-16 |
| Discussion | 23a | Provide a general interpretation of the results in the context of other evidence. | 11-15 |
|  | 23b | Discuss any limitations of the evidence included in the review. | 16 |
|  | 23c | Discuss any limitations of the review processes used. | 16 |
|  | 23d | Discuss implications of the results for practice, policy, and future research. | 11-13 |
| **OTHER INFORMATION** | | |  |
| Registration and protocol | 24a | Provide registration information for the review, including register name and registration number, or state that the review was not registered. | 4 |
|  | 24b | Indicate where the review protocol can be accessed, or state that a protocol was not prepared. | 4 |
|  | 24c | Describe and explain any amendments to information provided at registration or in the protocol. | 4 |
| Support | 25 | Describe sources of financial or non-financial support for the review, and the role of the funders or sponsors in the review. |  |
| Competing interests | 26 | Declare any competing interests of review authors. |  |
| Availability of data, code and other materials | 27 | Report which of the following are publicly available and where they can be found: template data collection forms; data extracted from included studies; data used for all analyses; analytic code; any other materials used in the review. |  |

***Appendix B:* Search Strategy**

The search keywords were [Title/Abstract]: posttraumatic stress disorder" OR "PTSD" OR "trauma" OR "panic disorder" OR "major depression" OR "severe depression" OR "depression" OR "major depressive disorder" OR "MDD" OR "anxiety disorder" OR "panic disorder" OR "agoraphobia" OR "phobia" OR "stress disorder" AND "Diffusion Tensor Imaging" OR "DIT" OR "Fractional anisotropy" AND "1980"[Date - Publication]: "2024/11/6"[Date - Publication]. These studies were included if: 1) performed whole-brain analysis, 2) included a comparison between patients with MDD/ANX/PTSD disorders and HC, 3) participants’ age was above 18 and below 65, 4) provided the t-map or coordinates in Montreal Neurological Institute [MNI]or Talairach space, 5) samples were free of any comorbid neurological conditions and 6) samples of patients had no or minimal current comorbid psychiatric disorders. To find the most optimal balance between excluding samples with any comorbidity (as they may be then nonrepresentative) and including highly comorbid samples, the maximum rate of current comorbidity with MDD/ANX/PTSD diagnosis was set at 25 %. If a sample was used in several studies, only the larger one was included. Similarly, only baseline data from longitudinal studies was incorporated.

**Search terms**

TS=( "posttraumatic stress disorder" OR "PTSD" OR "trauma" OR "panic disorder" OR "major depression" OR "severe depression" OR "depression" OR "major depressive disorder" OR "MDD"

**Embase**

('diffusion tensor imaging'):ab,ti OR ((DIT):ab,ti) OR (('Fractional anisotropy'):ab,ti)

('MDD'):ab,ti OR (('posttraumatic stress disorder'):ab,ti) OR (('PTSD'):ab,ti) OR (('trauma'):ab,ti) OR (('panic disorder'):ab,ti) OR (('major depression'):ab,ti) OR (('severe depression'):ab,ti) OR ((depression):ab,ti) OR (('major depressive disorder'):ab,ti)

**PubMed**

(("MDD"[Title/Abstract]) OR ("posttraumatic stress disorder"[Title/Abstract])) OR ("PTSD"[Title/Abstract])) OR ("trauma"[Title/Abstract])) OR ("panic disorder"[Title/Abstract])) OR ("major depression"[Title/Abstract])) OR ("severe depression"[Title/Abstract])) OR ("depression"[Title/Abstract])) OR ("major depressive disorder"[Title/Abstract]))

**SCOPUS**

( ( TITLE-ABS-KEY ( "Diffusion Tensor Imaging" ) OR TITLE-ABS-KEY ( "DIT" ) OR TITLE-ABS-KEY ( "Fractional anisotropy" ) ) ) AND ( ( TITLE-ABS-KEY ( "posttraumatic stress disorder" ) OR TITLE-ABS-KEY ( "PTSD" ) OR TITLE-ABS-KEY ( "trauma" ) OR TITLE-ABS-KEY ( "panic disorder" ) OR TITLE-ABS-KEY ( "major depression" ) OR TITLE-ABS-KEY ( "severe depression" ) OR TITLE-ABS-KEY ( "depression" ) OR TITLE-ABS-KEY ( "major depressive disorder" ) OR TITLE-ABS-KEY ( "MDD" )

**web of science（Web of Science Core Collection）**

TS=("posttraumatic stress disorder") OR TS=("PTSD") OR TS=("trauma") OR TS=("panic disorder") OR TS=("major depression") OR TS=("severe depression") OR TS=("depression") OR TS=("major depressive disorder") OR TS=("MDD")

**APA PsycInfo**

AB "posttraumatic stress disorder" OR AB "PTSD" OR AB trauma OR AB "panic disorder" OR AB "major depression" OR AB "severe depression" OR AB depression OR AB "major depressive disorder" OR AB "MDD"

***Appendix C:* Method of SDM-PSI**

Coordinates of significant clusters and their associated t-values were extracted when studies reported differences between the MDD, ANX, PTSD groups and the control group. If the original studies reported z-scores or p-values instead of t-values, these values were converted to t-values using the online tool available on the SDM Project website (sdmproject.com). When peak coordinates were reported in Talairach (TAL) space, they were converted into MNI space using matrix transformation. For each eligible study, an effect size-signed differential map was generated based on incorporated peaks extracted from the t-map or reported peak coordinates when the t-map was unavailable (Peters et al., 2012). When a statistical parametric map was available, the effect size (d) of each voxel was calculated using the sample size and t-value of that voxel. When there are no accessible t-maps, the effect size of peak voxel (dpeak) is firstly calculated and the effect size of remaining voxels is estimated by the close peaks(≤20mm) using the formula:d= dpeak *exp($-\frac{4*log(2)}{\mathrm{FWHM}}$*$D^{2}$), where FWHM is the full width at half maximum, *D* is the distance to close peaks(Radua et al., 2012).To allow for meta-analyzing of both VBA studies and TBSS studies, we adopted the TBSS template when generating the effect size-signed differential maps(Wise et al., 2016).The analysis utilized the FMRIB58 FA skeleton as a foundation, applied to both TBSS and VBA studies to ensure consistent coverage. The maps were preprocessed and assessed for consistency with the original manuscripts. A randomized effect size-signed differential image was subsequently generated using 20 whole-brain permutations, and a mean effect size map was calculated by aggregating the signed differential maps from each included study. The analytical processes adhered to the SDM-PSI tutorial, using parameters including anisotropy = 1.0, isotropic full-width at half maximum (FWHM) = 20 mm, peak height threshold = 1, voxel P = 0.005, and cluster extent = 10 voxels, with 10 repetitions of standard randomization tests. In addition, the SDM-PSI maps were overlaid onto a high-resolution brain image template from the International Consortium for Brain Mapping and visualized using MRIcron software.

***Appendix D: Age and gender differences***

1. All patients versus HCs

All patients, including MDD, ANX, PTSD (age=35.39±6.97; 58%females), were matched (*d*=0.175, *t*=0.967, *p*=0.513) with all corresponding HCs (age=34.24±6.15; 53% females) in age and sex (*d*=0.275, *t*=1.505, *p*=0.852). The results indicated no significant differences between the combined patient group (All diseases) and the combined HC group.

2. patients with MDD versus patients with ANX

There were no significant differences in age (*d* = -0.313, *t* = -0.915, *p* = 0.399), sex (*d* = 0.798, *t* = 2.14, *p* = 0.536), and symptom duration (*d* = 0.126, *t* = 0.261, *p* = 0.381) between patients with MDD and ANX.

3. Patients with MDD versus patients with PTSD

There were no differences regarding age (*d* = -0.743, *t* = -1.86, *p* = 0.979). However, participants with MDD (age = 34.42 ± 6.75; 61% females) had higher proportion of females (*d* = 0.405, *t* = 1.529, *p* < 0.001) than those with PTSD (age=39.18±6.04; 50% females). Sex was then included in the MDD vs PTSD meta-analysis as covariate.

4. Patients with ANX versus patients with PTSD

There were no differences regarding age (*d* = -0.339, *t* = -0.691, *p* = 0.445). However, participants with ANX (age = 36.75 ± 8.1; 50% females) differed significantly from those with PTSD in sex (*d* = 0.006, *t* = 0.013, *p* < 0.05). Sex was then included in the ANX vs PTSD meta-analysis as covariate.

5. Patients with MDD versus HC

There was no significant difference in age (*d* = 0.231, *t* = 1.085, *p* = 0.391) and sex between the patients with MDD and corresponding HCs; *d* = 0.456, *t* = 2.113, *p* = 0.703).

6. patients with ANX versus HC

Both age (*d*=0.225, *t* = 0.476, *p* = 0.472) and sex (*d* = 0.106, *t* = 0.224, *p* = 0.547) were found to be similar between the patients with ANX and corresponding HCs, with no statistically significant differences observed.

7. Patients with PTSD versus HC

The patients with PTSD were matched with all corresponding HCs (age=40.10±6.20; 50% females) in age (*d* = -0.151, *t* = -0.302, *p* = 0.813) and sex (*d* = 0.010, *t* = 0.020, *p* = 0.886). There was no significant difference between the PTSD group and the HC group.

8. patients with ANX and PTSD versus HC

There were no significant differences in age (*d* = 0.063, *t* = 0.184, *p* = 0.600) and sex (*d* = 0.034, *t* = 0.099, *p* = 0.939) between patients (with MDD and ANX) and corresponding HCs.

***Appendix E*: Anxiety disorders meta-analysis**

The meta-analysis comparing patients with anxiety disorder and PTSD to HC were exploratory analyses, So We applied a lenient threshold. Anxiety disorder meta-analysis revealed significant FA reductions in the right inferior network and inferior frontal-occipital fasciculus in ANX compared with HC (*p* < 0.1, uncorrected), This finding aligns with the results of earlier research studies. Including Generalized anxiety disorder(M. Liao et al., 2014), Panic disorder(Lai & Wu, 2013), Trait anxiety(Baur et al., 2012), and Social anxiety disorder(Baur et al., 2011).The inferior frontal-occipital fasciculus (IFOF) is one of the first major association fiber systems recognized and depicted in the human brain(Schmahmann & Pandya, 2007). Our findings indicate a decrease in FA in the inferior frontal-occipital fasciculus. The IFOF connects the occipital cortex, temporal-basal areas, and superior parietal lobe to the frontal lobe via the depth of the temporal lobe and insula(Martino et al., 2010). It is thought to be responsible for connecting the frontal and occipital lobes and regulating how we respond to environmental stimuli that cause anxiety, Besides, frontal lobes and sensory-related regions (e.g., occipital lobes) are important components of the fear network model (Gorman & Sullivan, 2000). Decreased FA in the inferior frontal-occipital fasciculus may disrupt communication between the occipital and frontal lobes, resulting in impaired sensory integration and cognitive or emotional regulation in response to sensory stimuli(M. Liao et al., 2014). Our meta-analysis proposes that alterations to the IFOF due to FA may impair sensory integration and cognitive inhibition of sensory stimuli and emotions, which could be linked to the pathophysiology of anxiety.

***Appendix F*: Funnel plot**

**1. ANX+PTSD<HC**


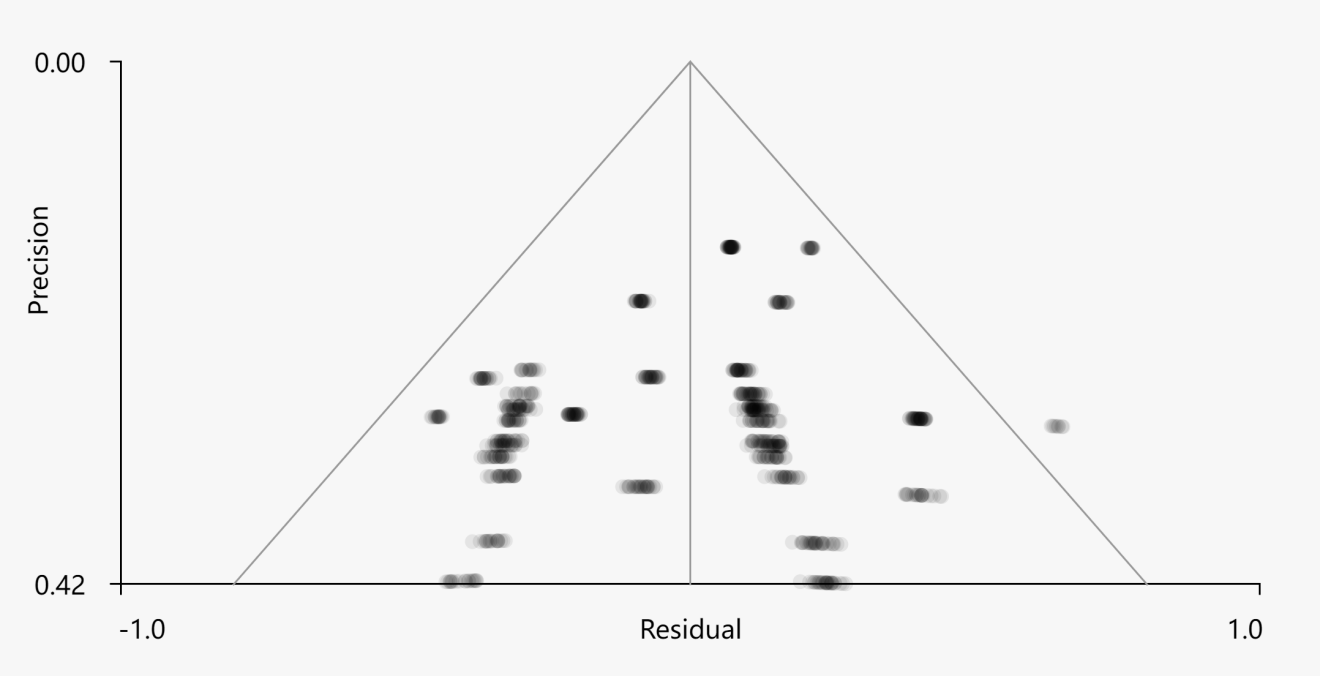


**Pe****ak2 : -22,50,22 Bias: -0.56, z: -0.52, df: 15, p: 0.602**


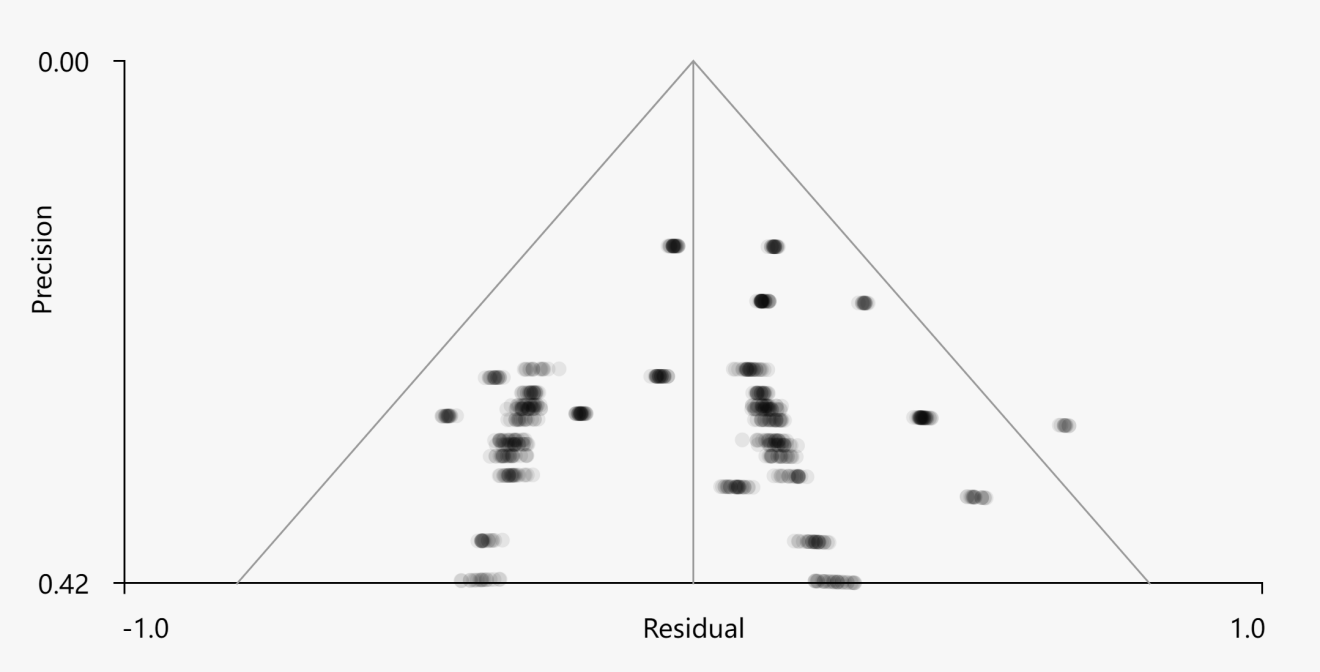


**Peak4: -30,50,26 Bias: -0.46, z: -0.40, df: 15, p: 0.693**


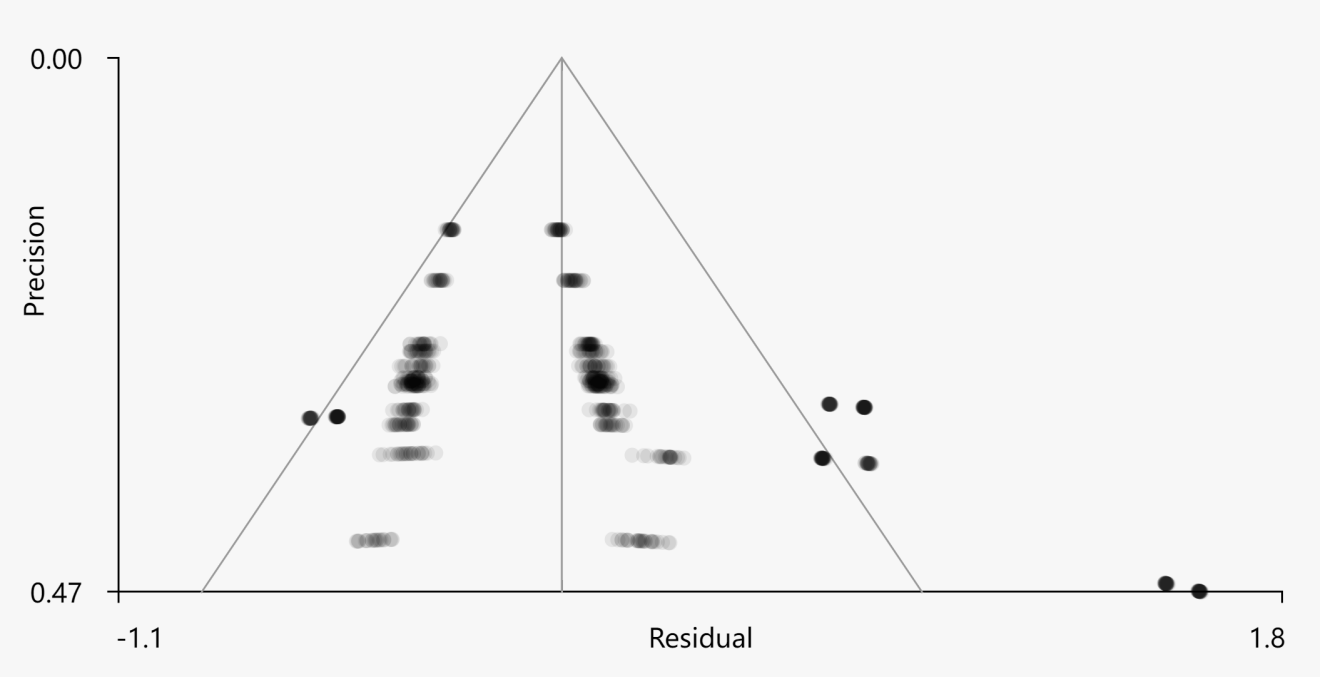


**Peak3: 4,-4,-2 Bias: 2.79, z: 1.79, df: 15, p: 0.073**

**2. MDD<HC**


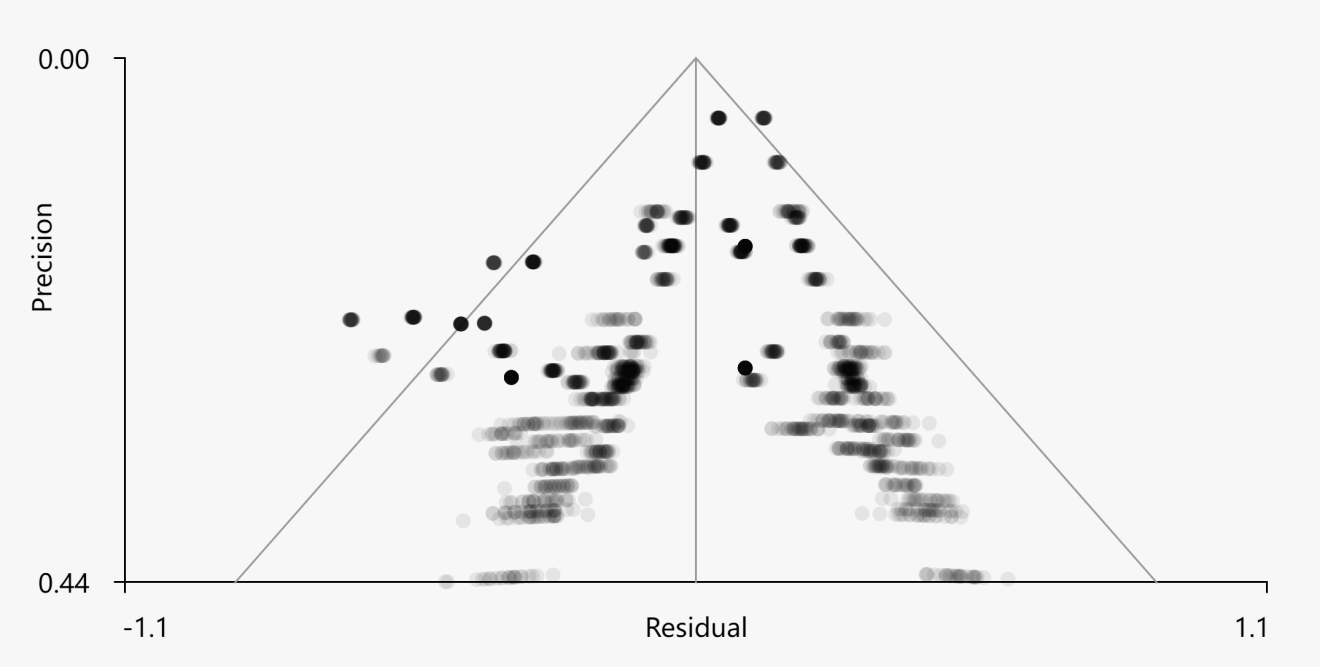


**Peak1 : -6,16,18 Bias: -0.37, z: -0.81, df: 42, p: 0.419**


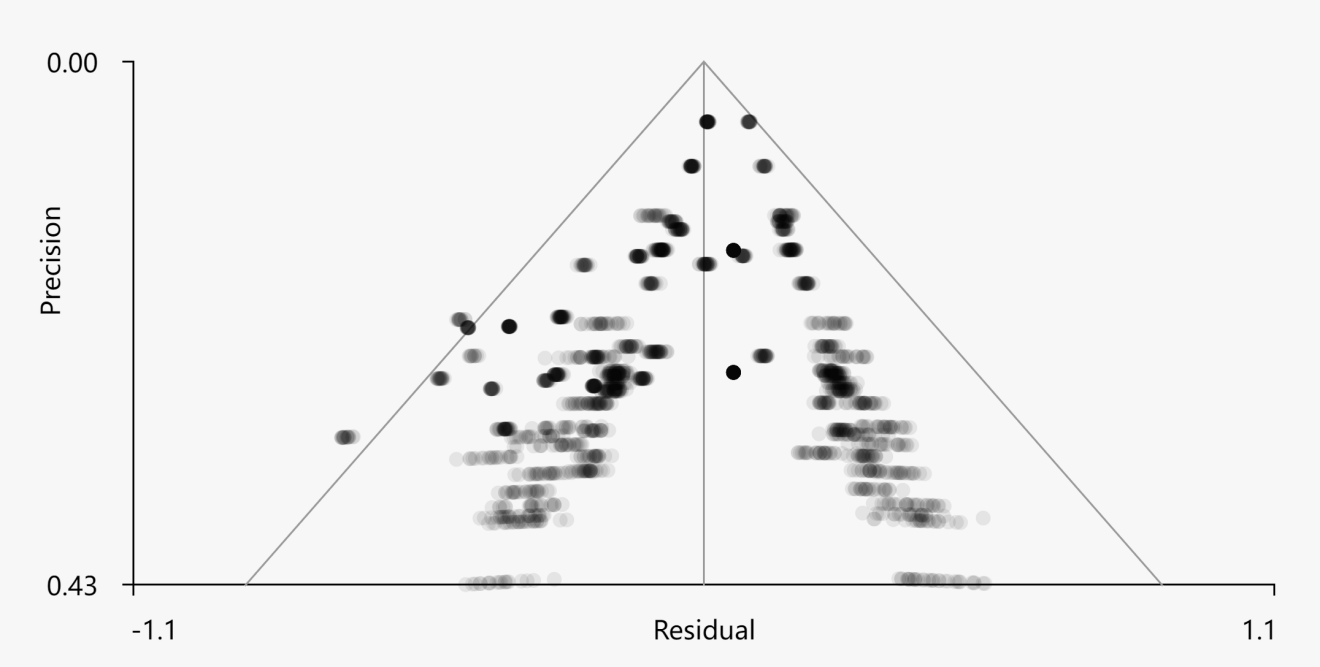


**Peak2: -2,2,24 Bias: -0.33, z: -0.92, df: 42, p: 0.360**


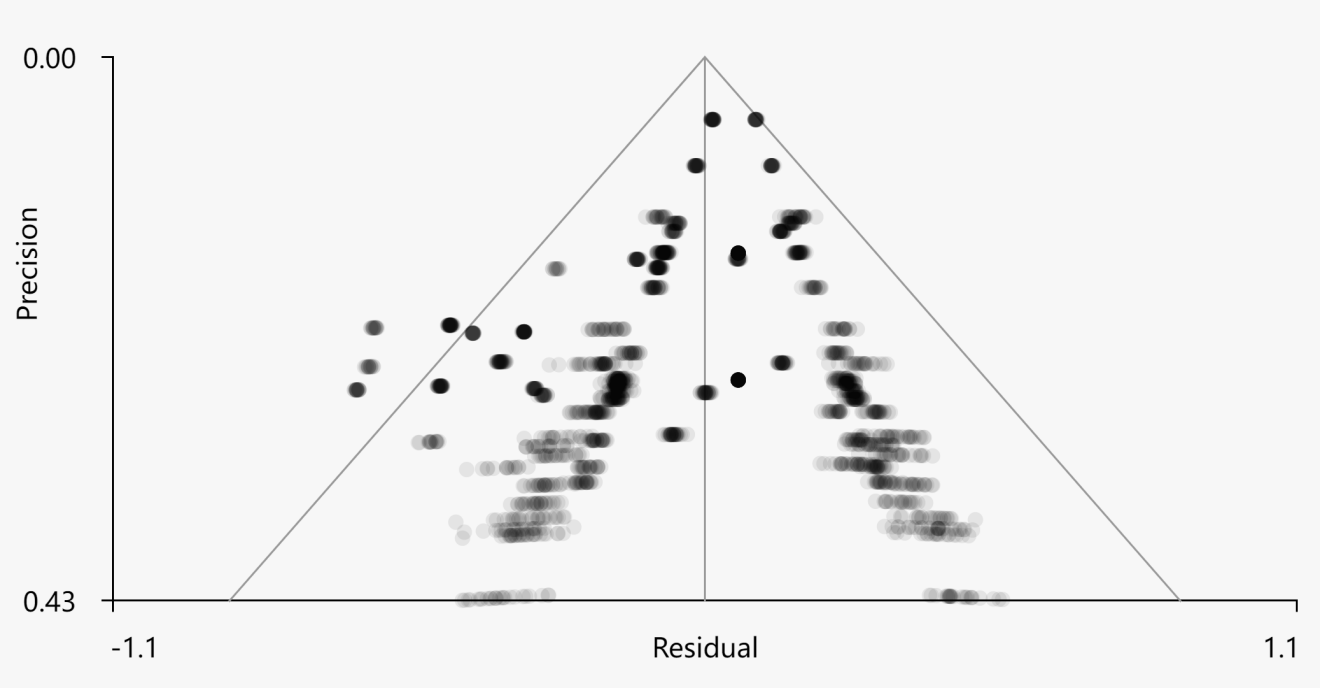


**Peak3: 0,6,18 Bias: -0.35, z: -0.90, df: 42, p: 0.368**


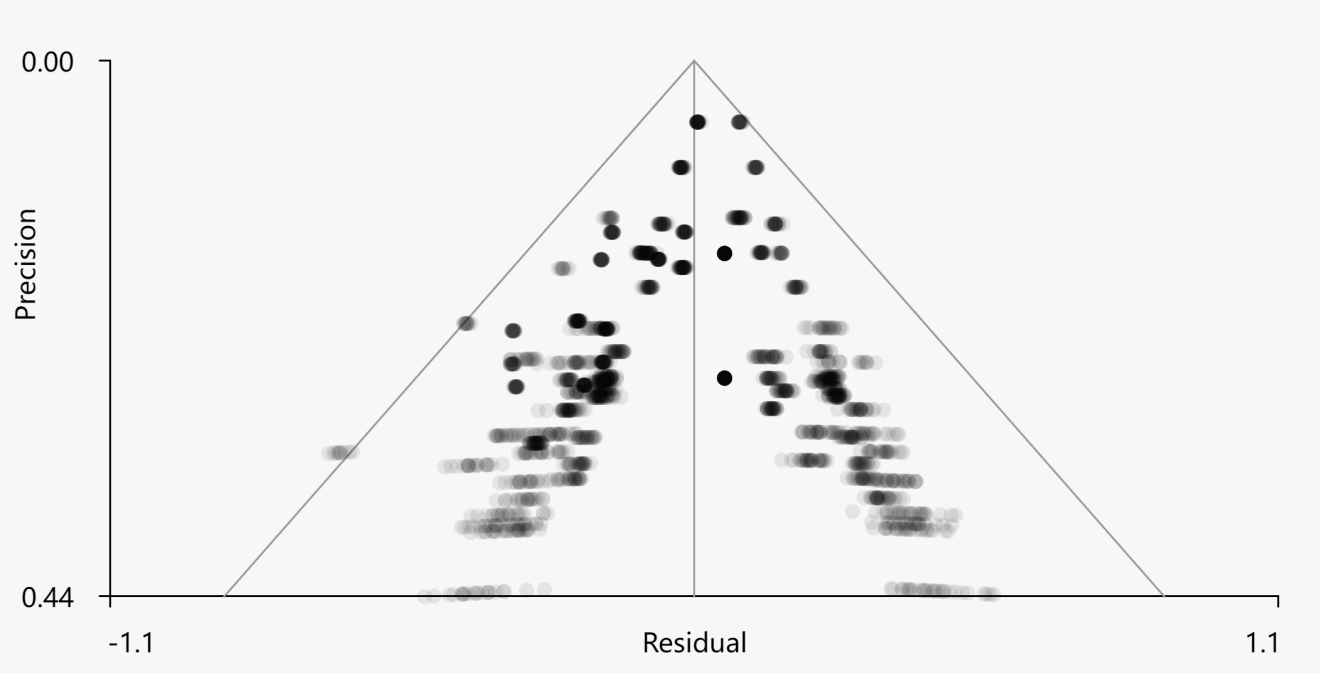


**Peak4:-20,14,20 Bias: -0.22, z: -0.59, df: 42, p: 0.558**

**3. MDD+PTSD+ANX<HC**


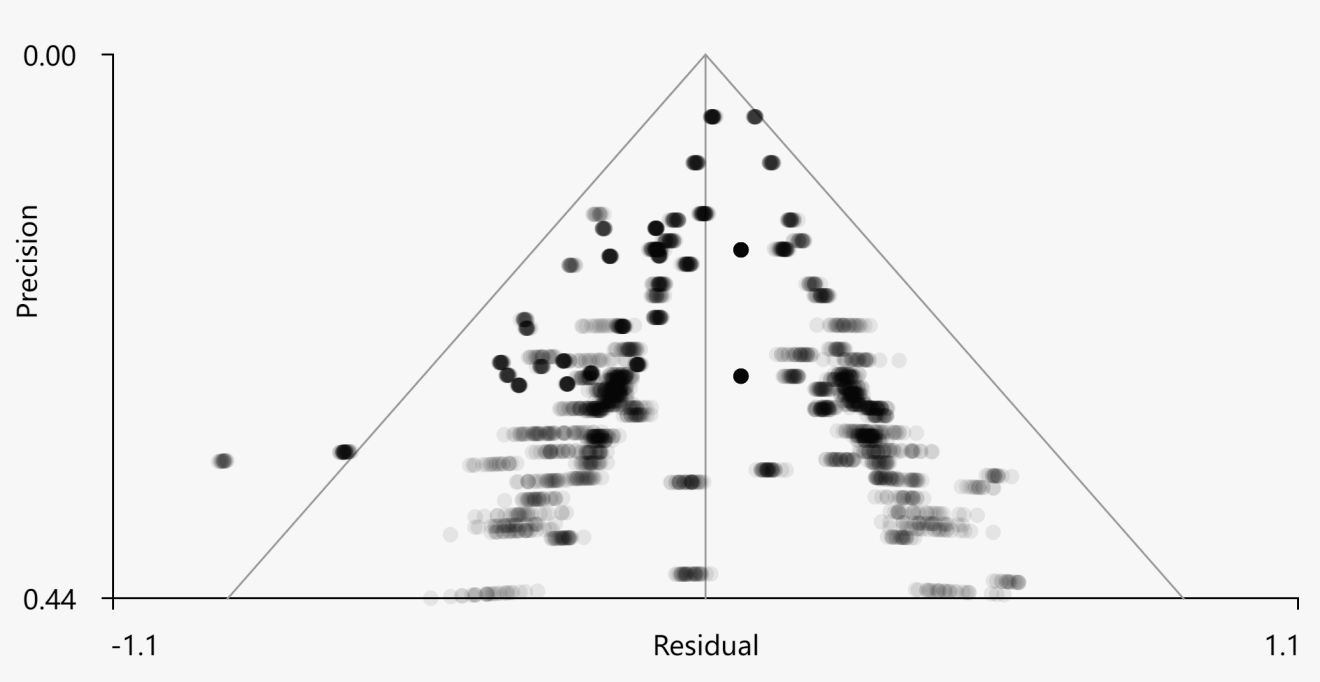


**Peak1: -20,24,18 Bias: -0.17, z: -0.55, df: 59, p: 0.585**


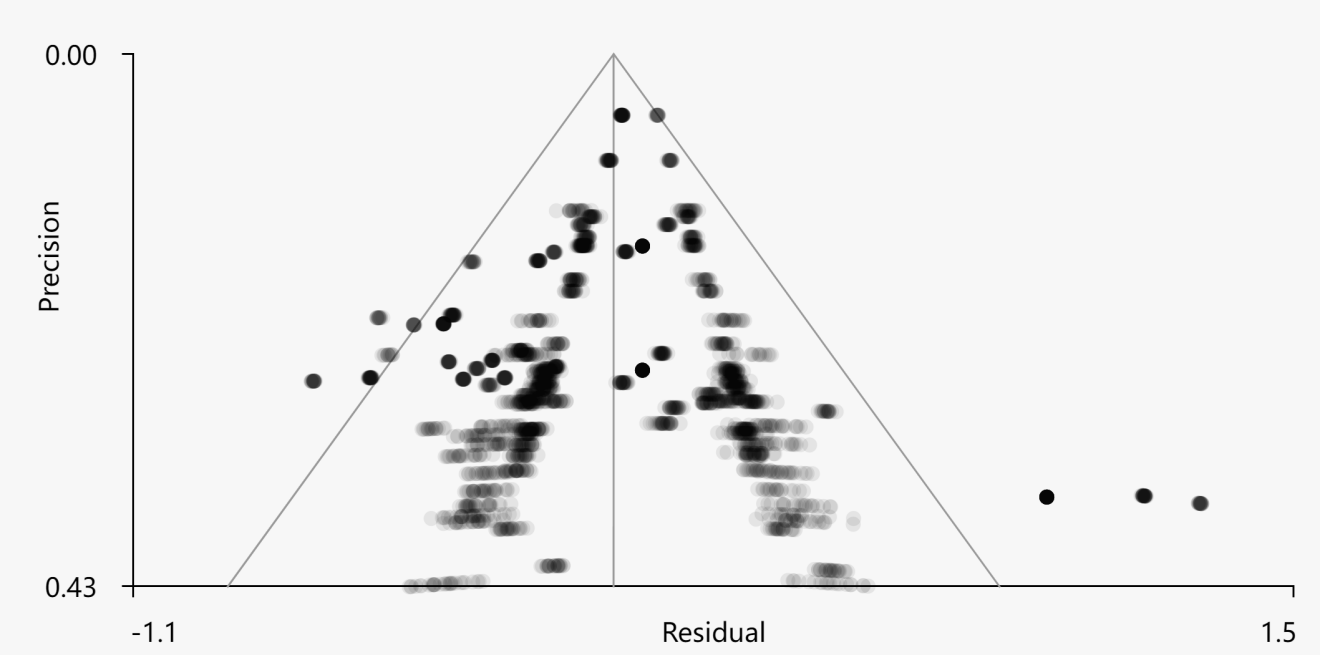


**Peak2: 0,12,20 Bias: 0.10, z: 0.24, df: 59, p: 0.810**


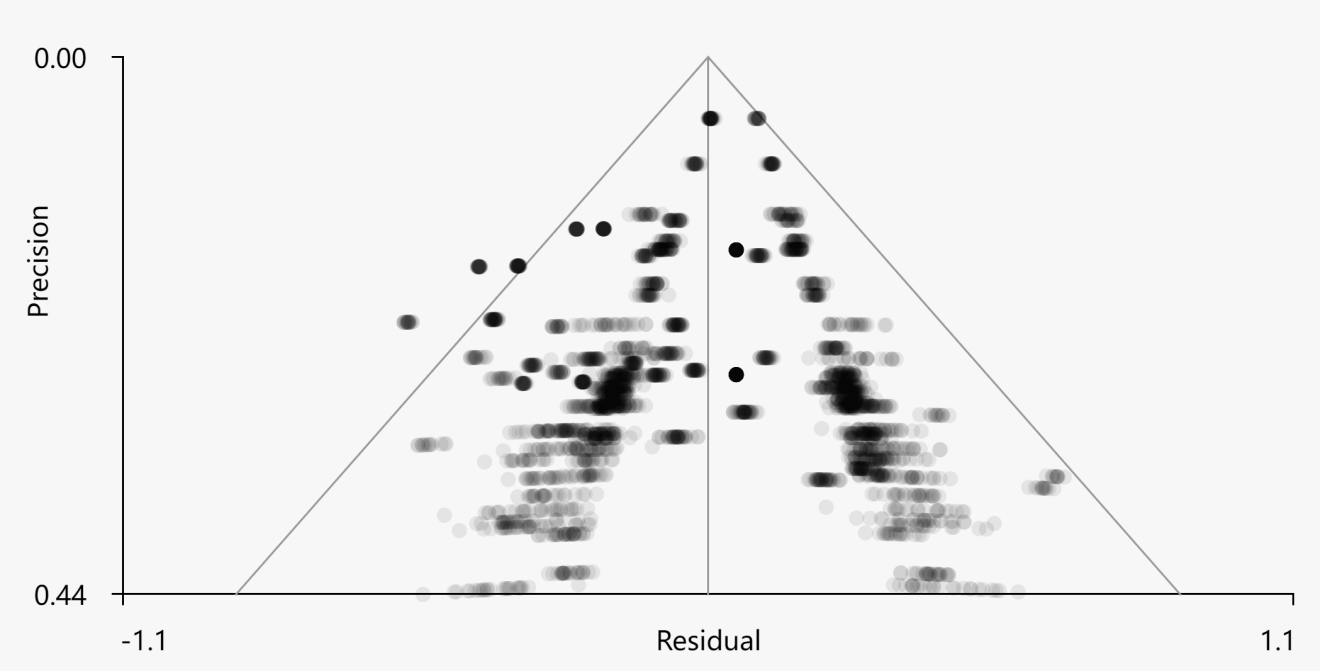


**Peak3：-10,30,8 Bias: -0.09, z: -0.30, df: 59, p: 0.761**


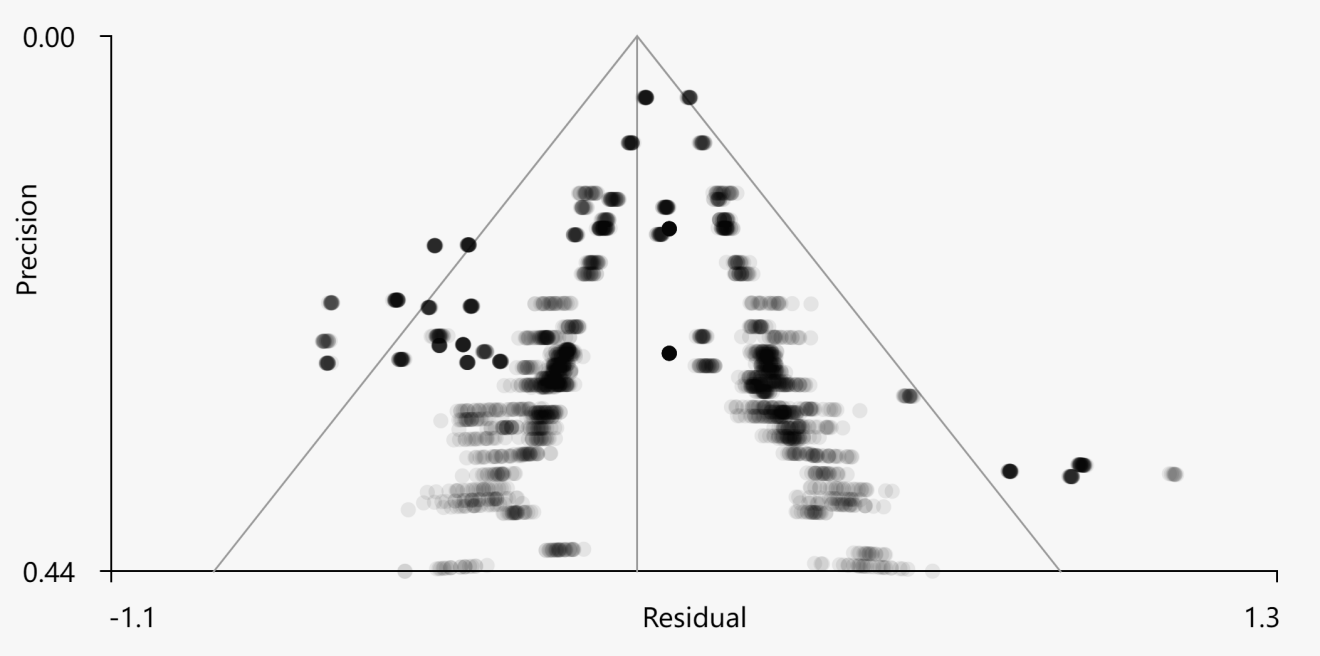


**Peak4: -2,18,16 Bias: 0.15, z: 0.33, df: 59, p: 0.739**

**4. MDD<ANX**


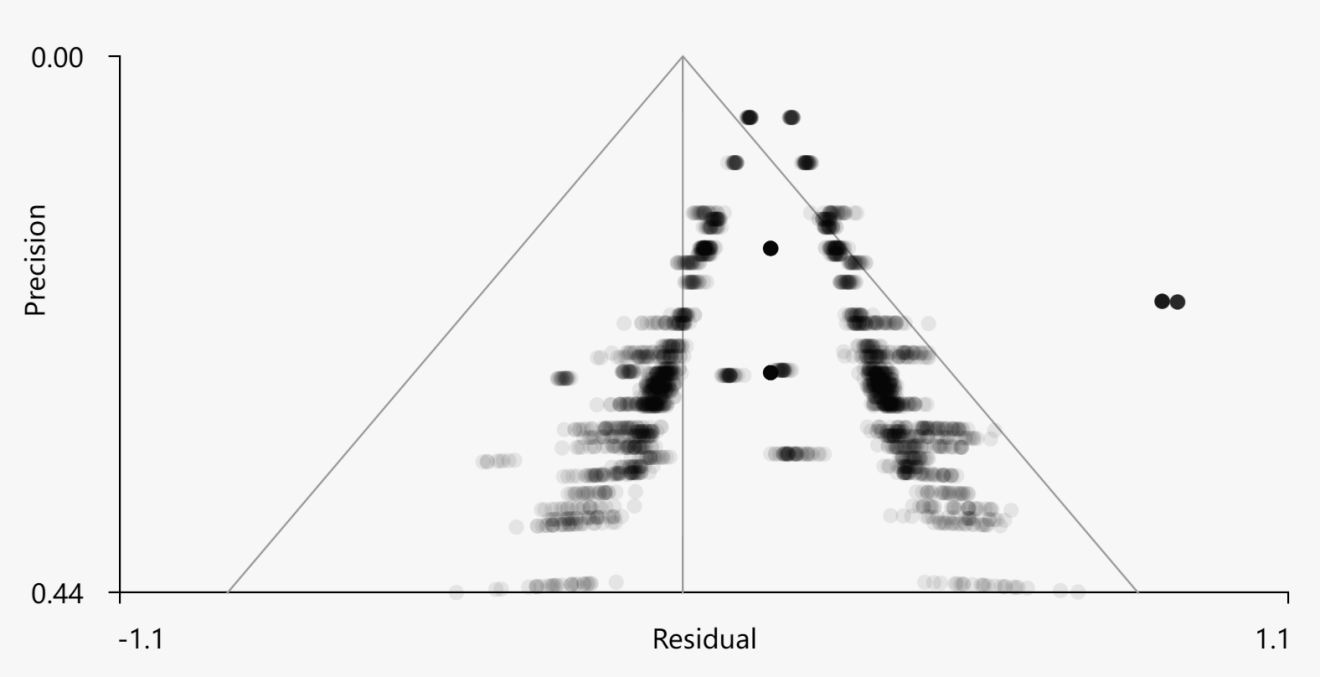


**Peak1: 26,-58,-40 Bias: -0.18, z: -0.62, df: 51, p: 0.536**


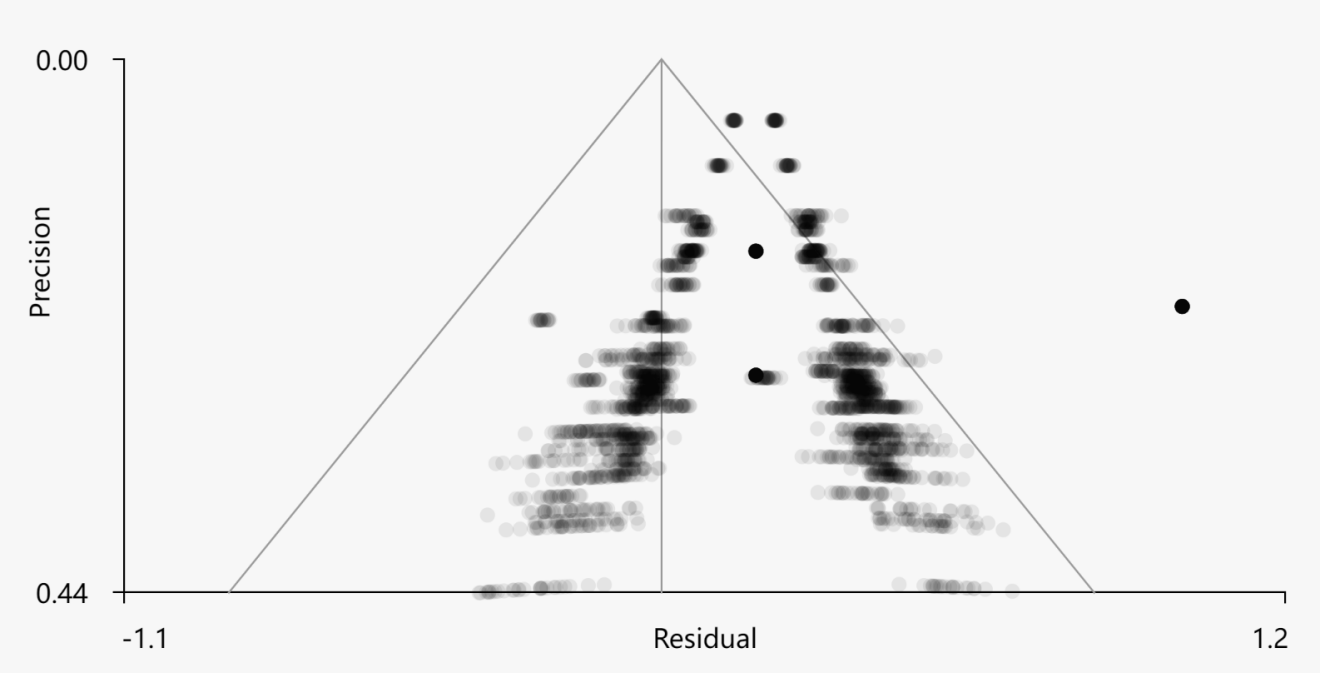


**Peak2: -30,-44,34 Bias: -0.21, z: -0.71, df: 51, p: 0.479**


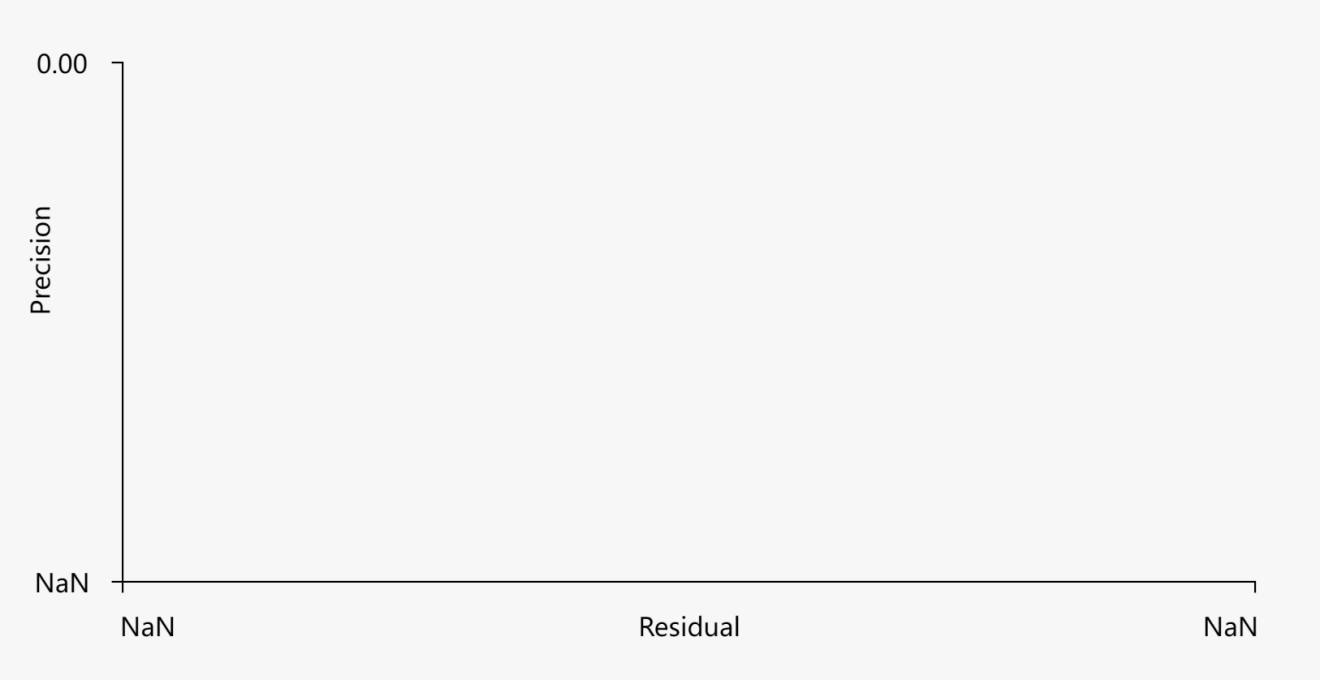


**Peak3: 32,-46,34 Bias: nan, z: nan, df: 51, p: nan**

**5. MDD<PTSD**


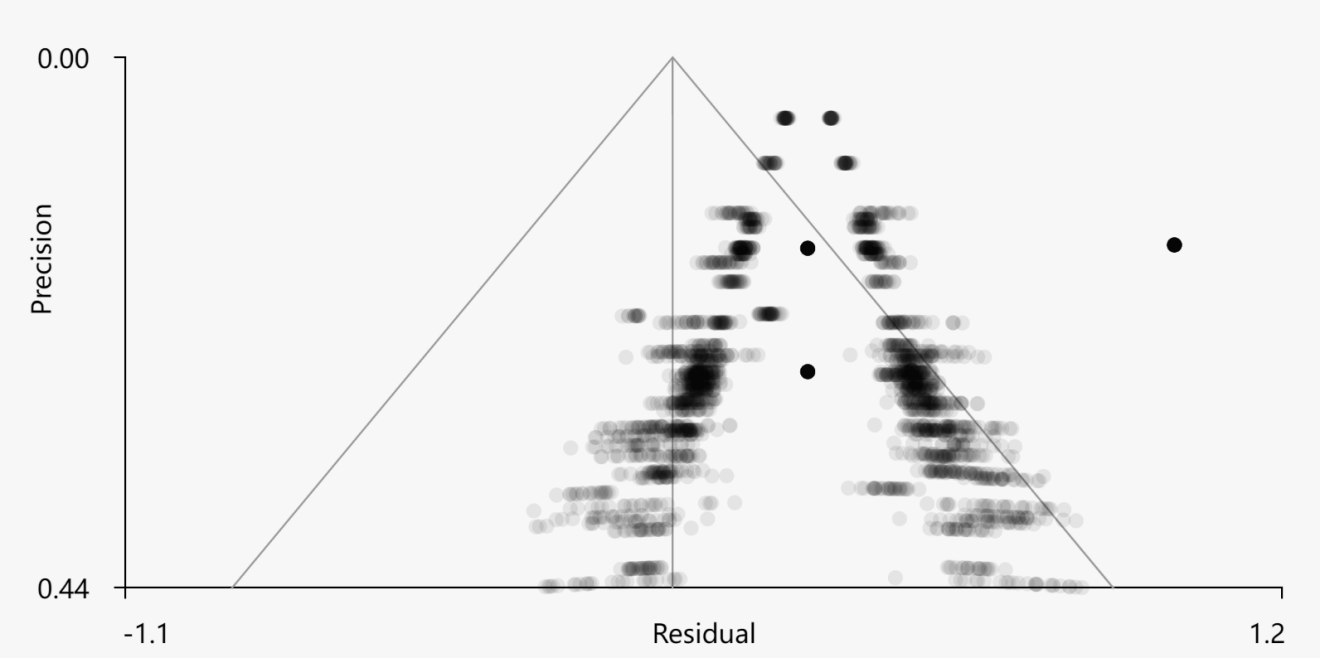


**Peak1: -24,-60,14 Bias: -0.28, z: -0.94, df: 50, p: 0.346**


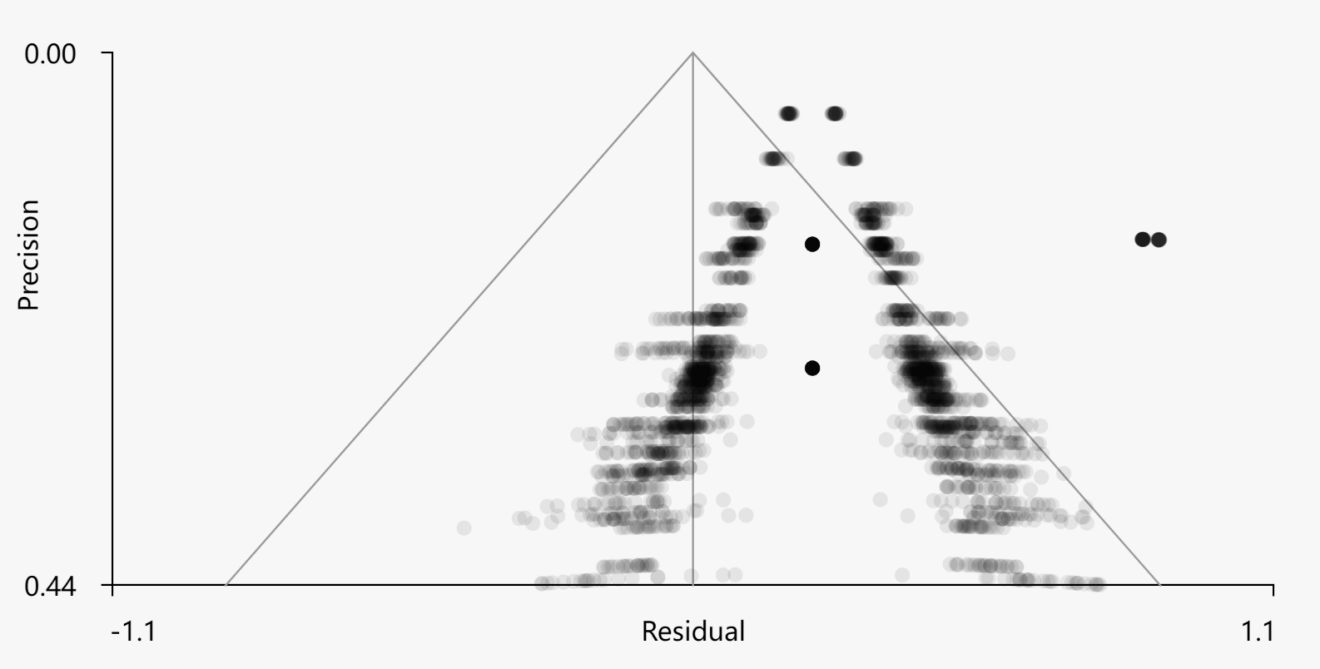


**Peak2: -34,46,6Bias: -0.24, z: -0.83, df: 50, p: 0.405**


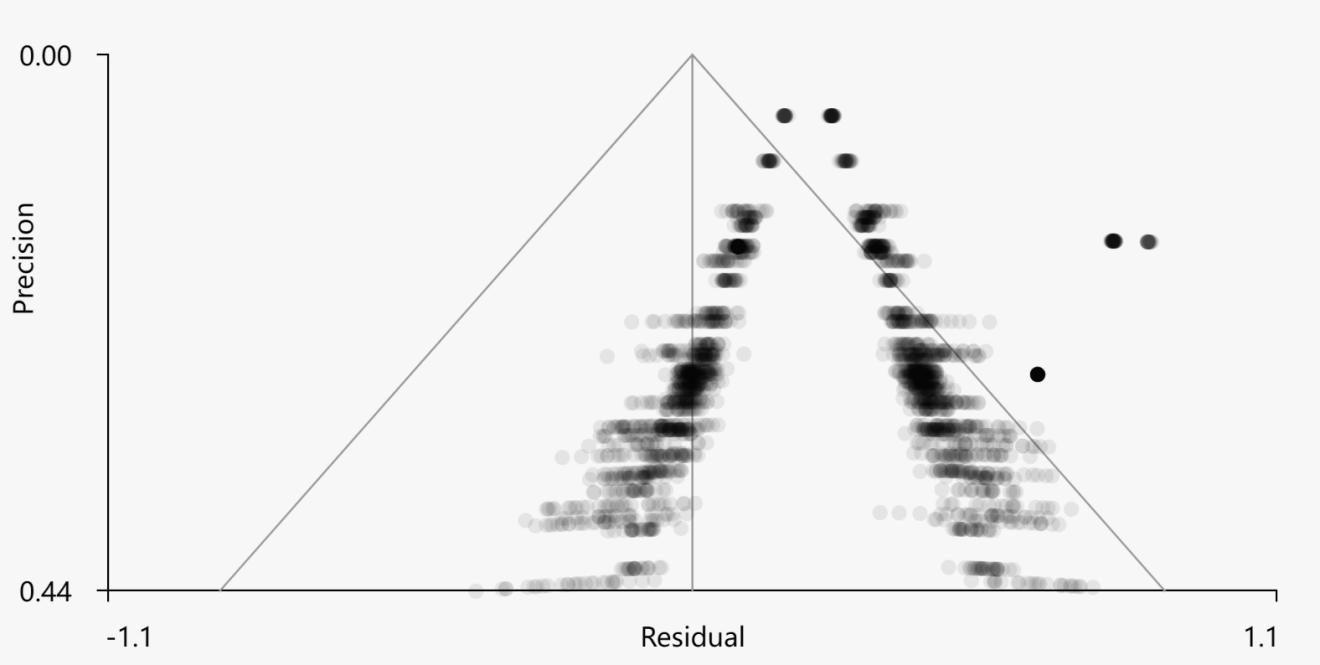


**Peak3: -20,50,2Bias: -0.23, z: -0.79, df: 50, p: 0.429**

**6. PTSD<HC**


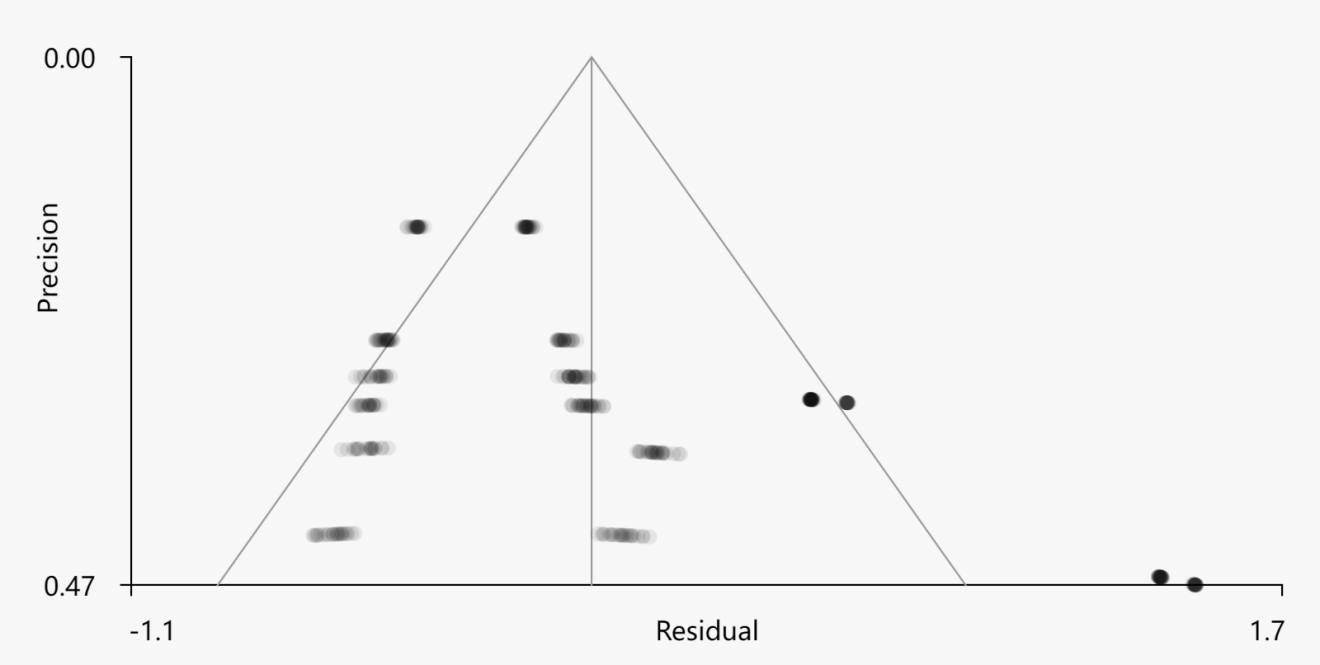


**Peak2: 4,-4,-2Bias: 3.14, z: 1.51, df: 6, p: 0.132**

**7.** **Subgroup analysis**

**MDD+ANX+PTSD:NO-MEDICINE**


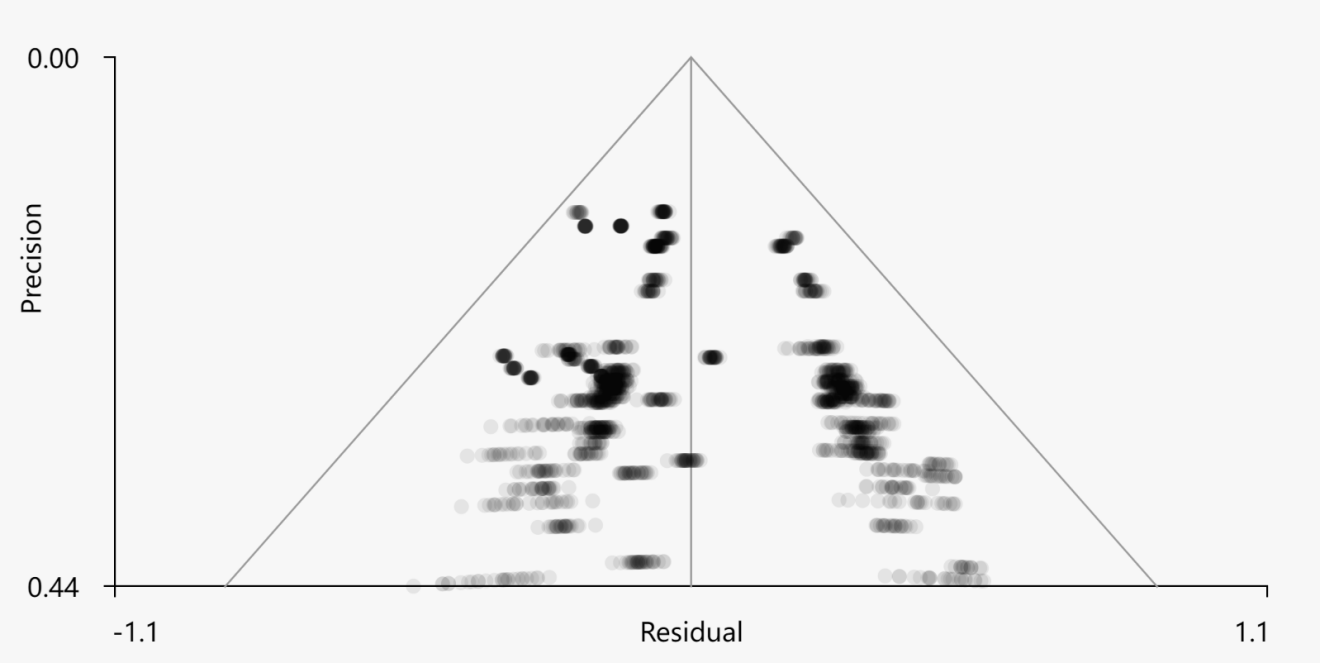


**Peak2: -22,34,8Bias: 0.46, z: 0.72, df: 40, p: 0.472**

**MDD+ANX+PTSD: TBSS**


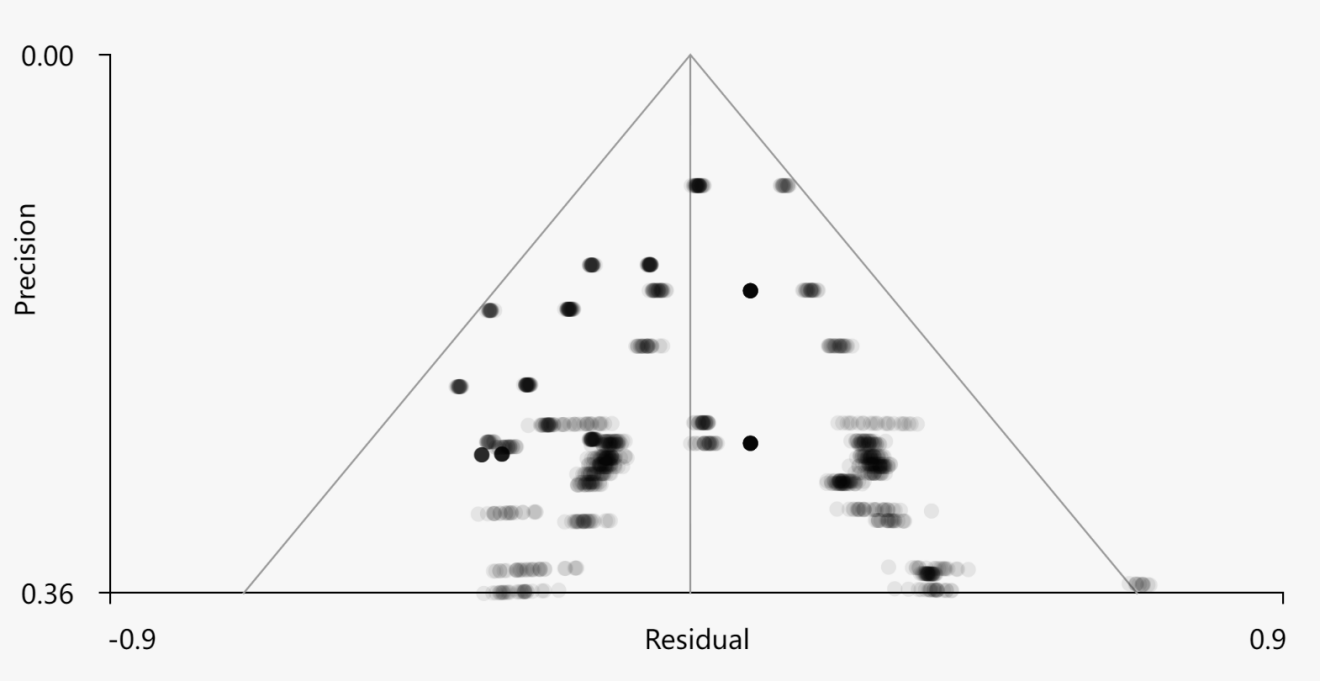


**Peak1: -12,20,20Bias: 0.06, z: 0.09, df: 25, p: 0.925**


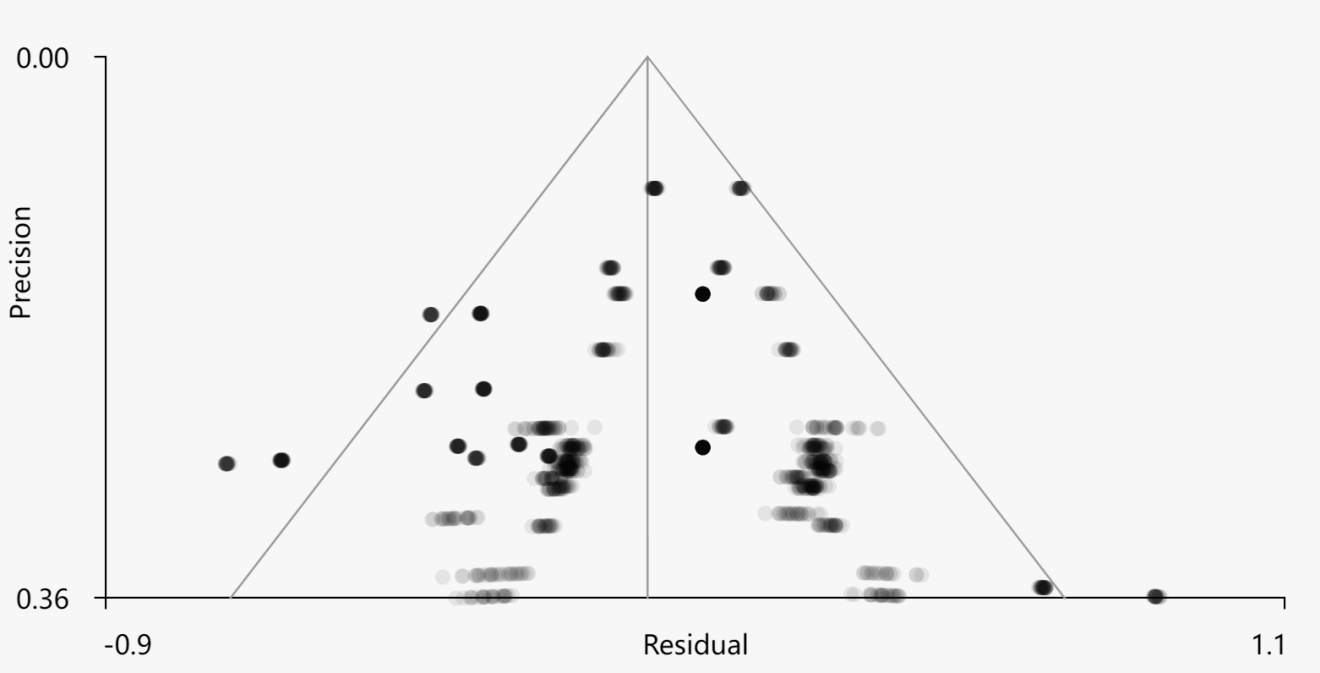


**Peak2: 2,18,16Bias: 0.09, z: 0.11, df: 25, p: 0.911**


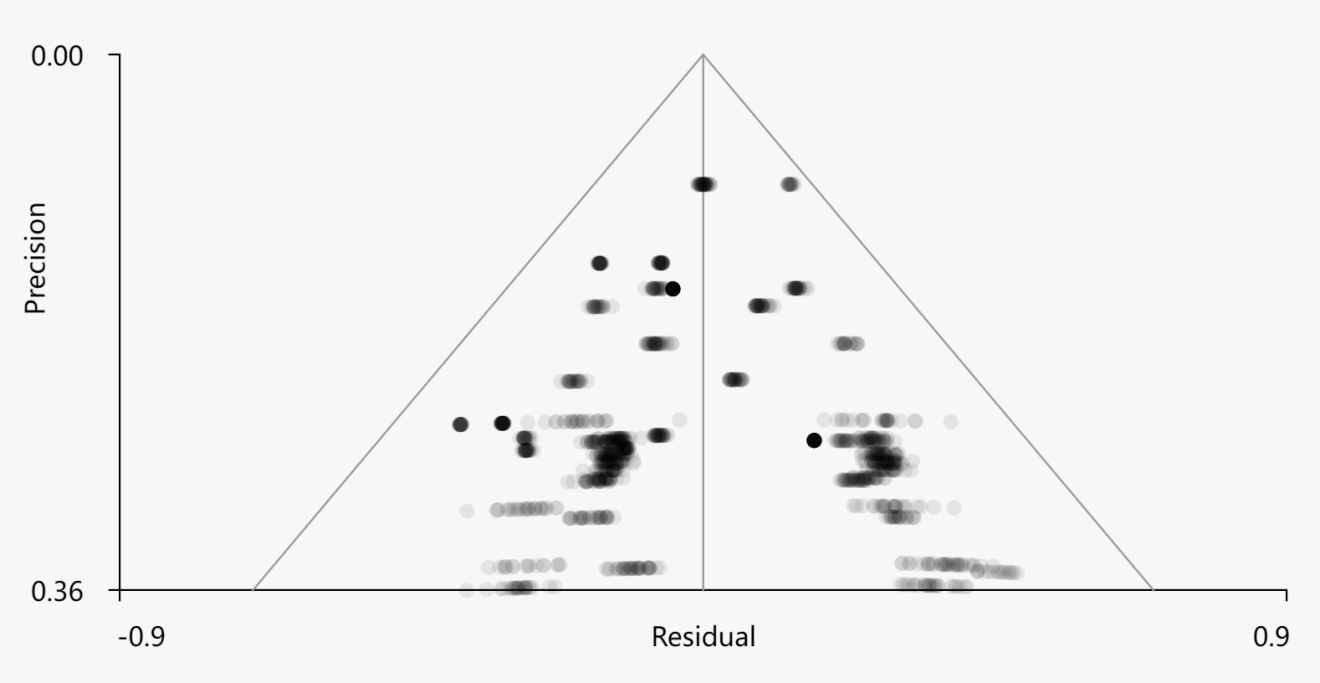


**Peak3: -20,32,16Bias: 0.06, z: 0.09, df: 25, p: 0.927**

**MDD+ANX+PTSD: VBA**


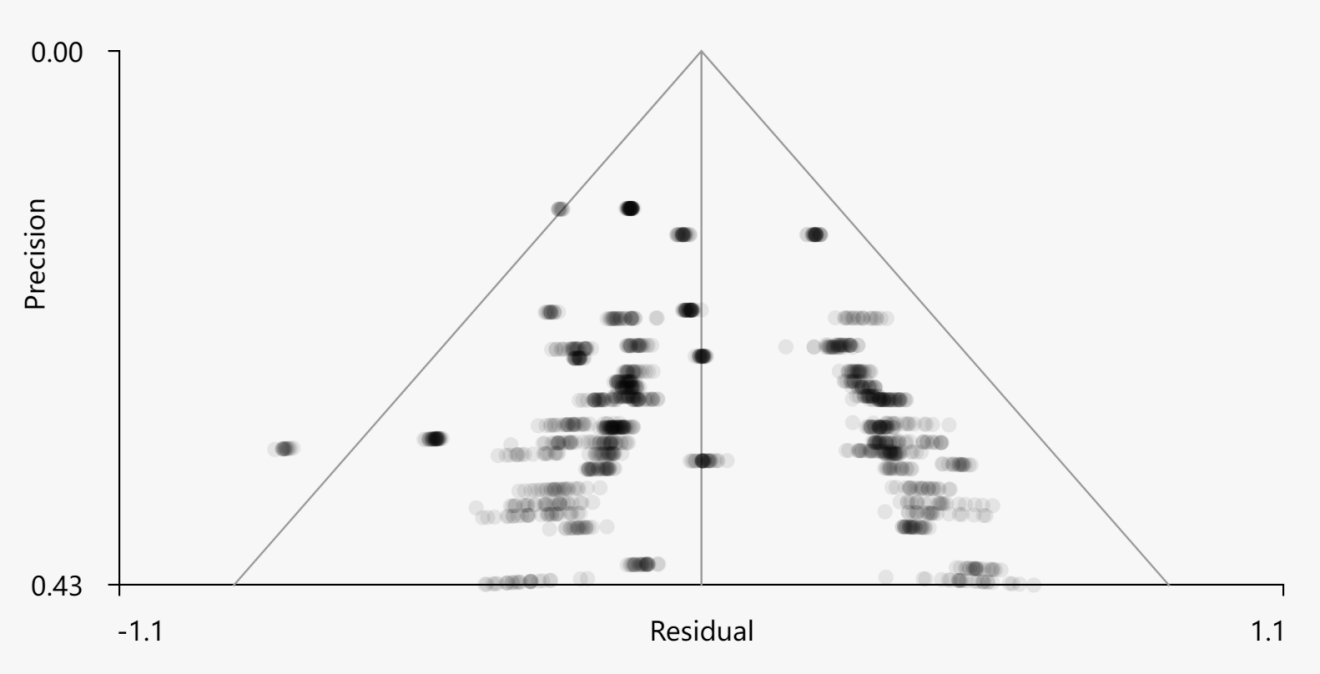


**Peak1: -24,26,10Bias: 0.47, z: 0.60, df: 27, p: 0.549**

***Appendix G*: Coordinates for each result:**

Table1: MDD<HC

| Region | MNI | SDM-Z | P value | voxels | I^2^ | Effect size | Metabias test p |  |
| --- | --- | --- | --- | --- | --- | --- | --- | --- |
| 1. Corpus callosum Left IN. IFOF | **-6,16,18** | **-2.431** | **0.007521033** | **241** | **13.044** | **-0.092** | **0.419** |  |
| Corpus callosum | 0,12,20 | -2.391 | 0.008391678 |  |  |  |  |  |
| Corpus callosum | -20,32,16 | -2.281 | 0.011271179 |  |  |  |  |  |
| Corpus callosum | -2,18,16 | -2.272 | 0.011546850 |  |  |  |  |  |
| Corpus callosum | -20,24,18 | -2.179 | 0.014661789 |  |  |  |  |  |
| Corpus callosum | -20,36,10 | -2.177 | 0.014735341 |  |  |  |  |  |
| Corpus callosum | -12,18,22 | -2.114 | 0.017269254 |  |  |  |  |  |
| Corpus callosum | -12,22,18 | -2.080 | 0.018761158 |  |  |  |  |  |
| Corpus callosum | 4,12,20 | -1.985 | 0.023592174 |  |  |  |  |  |
| Corpus callosum | -14,30,12 | -1.919 | 0.027505577 |  |  |  |  |  |
| Corpus callosum | 4,8,22 | -1.863 | 0.031214178 |  |  |  |  |  |
| 2. Corpus callosum | **-2,2,24** | **-1.748** | **0.040189505** | **5** | **0.031** | **-0.056** | **0.360** |  |
| 3. Corpus callosum (undefined) | | **0,6,18** | **-1.783** | **0.037317514** | **2** | **2.182** | **-0.060** | **0.368** |
| 4. Left ATP | **-20,14,20** | **-1.771** | **0.038279176** | **2** | **0.000** | **-0.056** | **0.558** |  |

Left IN. IFOF: Left inferior network, inferior fronto-occipital fasciculus; Left ATP: Left anterior thalamic projections

Table2:MDD+PTSD+ANX<HC

| Region | MNI | SDM-Z | P value | voxels | I^2^ | Effect size | Metabias test p |  |
| --- | --- | --- | --- | --- | --- | --- | --- | --- |
| 1. Corpus callosum Left IN. IFOF | **-20,24,18** | **-2.193** | **0.014167130** | **166** | **0.000** | **-0.064** | **0.585** |  |
| Corpus callosum | -20,32,16 | -2.187 | 0.014388442 |  |  |  |  |  |
| Corpus callosum | -20,36,10 | -2.122 | 0.016936839 |  |  |  |  |  |
| Corpus callosum | -12,18,22 | -2.047 | 0.020320058 |  |  |  |  |  |
| Left ATP | -22,32,12 | -2.041 | 0.020625353 |  |  |  |  |  |
| Left striatum | -24,24,16 | -2.031 | 0.021123230 |  |  |  |  |  |
| Corpus callosum | -4,24,12 | -1.908 | 0.028168023 |  |  |  |  |  |
| Corpus callosum | -8,18,18 | -1.904 | 0.028450191 |  |  |  |  |  |
| Corpus callosum | -14,30,12 | -1.860 | 0.031462371 |  |  |  |  |  |
| Corpus callosum | -16,30,16 | -1.841 | 0.032792628 |  |  |  |  |  |
| Left striatum | -24,20,16 | -1.822 | 0.034252048 |  |  |  |  |  |
| Left ATP | -20,14,20 | -1.798 | 0.036076725 |  |  |  |  |  |
| Corpus callosum | -18,16,28 | -1.707 | 0.043934822 |  |  |  |  |  |
| Corpus callosum | -10,12,26 | -1.707 | 0.043947160 |  |  |  |  |  |
| 2. Corpus callosum | **0,12,20** | **-1.788** | **0.036890864** | **1** | **15.124** | **-0.064** | **0.810** |  |
| 3. Corpus callosum | | **-10,30,8** | **-1.679** | **0.046559989** | **1** | **0.015** | **-0.052** | **0.761** |
| 4. Corpus callosum | **-2,18,16** | **-1.649** | **0.049552143** | **1** | **20.090** | **-0.065** | **0.739** |  |

Left IN. IFOF: Left inferior network, inferior fronto-occipital fasciculus; Left ATP: Left anterior thalamic projections

Table3:PTSD<HC

| Region | MNI | SDM-Z | P value | voxels | I^2^ | Effect size | Metabias test p |
| --- | --- | --- | --- | --- | --- | --- | --- |
| 1. Right ATP | **4,-4,-2** | **1.514** | **0.045032601** | **9** | **58.099** | **0.298** | **0.132** |

Right ATP: Right anterior thalamic projections

Table4:MDD<ANX

| Region | MNI | SDM-Z | P value | voxels | I^2^ | Effect size | Metabias test p |  |  |
| --- | --- | --- | --- | --- | --- | --- | --- | --- | --- |
| 1.MCP(undefined) | **26,-58,-40** | **-1.534** | **0.042494099** | **38** | **0.000** | **-0.166** | **0.536** |  |  |
| (undefined) | 20,-58,-38 | -1.527 | 0.043404560 |  |  |  |  |  |  |
| MCP | 24,-54,-40 | -1.453 | 0.053062539 |  |  |  |  |  |  |
| 2. Left SLF II  Corpus callosum | **-30,-44,34** | **-1.638** | **0.040686836** | **22** | **0.179** | **-0.187** | **0.479** |  |  |
| Left SLF II | | | -28,-40,30 | -1.492 | 0.047784607 |  |  |  |  |
| Corpus callosum | | | -28,-44,26 | -1.312 | 0.044683647 |  |  |  |  |
| 3. Right SLF II | | **32,-46,34** | **-1.622** | **0.042374482** | **10** | **0.003** | **-0.183** | **NA** |  |
| 4. Corpus callosum | **-28,-36,26** | **-1.455** | **0.042803199** | **8** |  |  |  |  |  |
| Corpus callosum | -26,-32,24 | -1.437 | 0.045415373 |  |  |  |  |  |  |
| Corpus callosum | -28,-34,20 | -1.293 | 0.058066926 |  |  |  |  |  |  |

MCP: Middle cerebellar peduncles; Left SLF :Left superior longitudinal fasciculus; Right SLF : Right superior longitudinal fasciculus

Table5: MDD<PTSD

| Region | MNI | SDM-Z | P value | voxels | I^2^ | Effect size | Metabias test p |
| --- | --- | --- | --- | --- | --- | --- | --- |
| 1. Corpus callosum | **-24,-60,14** | **-2.153** | **0.015648901** | **20** | **0.636** | **-0.262** | **0.346** |
| 2. Left ATP | **-34,46,6** | **-1.958** | **0.025139570** | **3** | **0.000** | **-0.219** | **0.405** |
| 3. Corpus callosum | **-20,50,2** | **-1.798** | **0.036080956** | **2** | **0.028** | **-0.211** | **0.429** |

Left ATP: Left anterior thalamic projections

Table6: ANX+PTSD<HC

| Region | | MNI | | SDM-Z | | P value | | voxels | | I^2^ | | Effect size | | Egger test p |  |  |
| --- | --- | --- | --- | --- | --- | --- | --- | --- | --- | --- | --- | --- | --- | --- | --- | --- |
| 1. Corpus callosum | | **-22,50,22** | | **1.521** | | **0.044179599** | | **27** | | **0.054** | | **0.118** | | ***0.602*** |  |  |
| Corpus callosum | | -32,56,16 | | 1.471 | | 0.040664763 | |  | |  | |  | |  |  |  |
| Corpus callosum | | -26,56,18 | | 1.467 | | 0.051188509 | |  | |  | |  | |  |  |  |
| Corpus callosum | | -26,50,24 | | 1.416 | | 0.058315854 | |  | |  | |  | |  |  |  |
| Corpus callosum | | -30,50,16 | | 1.406 | | 0.059796433 | |  | |  | |  | |  |  |  |
| 2. Corpus callosum | | **-30,50,26** | | **1.457** | | **0.042580338** | | **6** | | **0.279** | | **0.108** | | ***0.693*** |  |  |
| Left SLF II | | | | | | | |  | |  | |  | |  |  |  |
| Left SLF II | | | -30,46,28 | | 1.393 | | 0.051769228 | |  | |  | |  | |  |  |
| 3. Right ATP | | | **4,-4,-2** | | **1.370** | | **0.045388422** | | **2** | | **55.909** | | **0.153** | | ***0.073*** | |

Left SLF II : Left superior longitudinal fasciculus II; Right ATP: Right anterior thalamic projections

Table7: **MDD+ANX+PTSD:NO-MEDICINE**

| Region | MNI | SDM-Z | P value | voxels | I^2^ | Effect size | Metabias test p |
| --- | --- | --- | --- | --- | --- | --- | --- |
| 1. Corpus callosum Left striatum  Left ATP | **-22,34,8** | **-1.758** | **0.039360583** | **20** | **0.000** | **-0.079** | **0.472** |
| Left ATP | -24,30,12 | -1.736 | 0.041318238 |  |  |  |  |
| Left ATP | -22,26,16 | -1.718 | 0.042932630 |  |  |  |  |
| Corpus callosum | -20,32,16 | -1.705 | 0.044115901 |  |  |  |  |

Left ATP: Left anterior thalamic projections

Table8: **MDD+ANX+PTSD: TBSS**

| Region | MNI | SDM-Z | P value | voxels | I^2^ | Effect size | Metabias test p |
| --- | --- | --- | --- | --- | --- | --- | --- |
| 1.Corpus callosum Left ATP | **-12,20,20** | **-2.087** | **0.018433928** | **67** | **0.046** | **-0.095** | **0.925** |
| Corpus callosum | -10,26,14 | -2.050 | 0.020203829 |  |  |  |  |
| Corpus callosum | -20,24,18 | -1.923 | 0.027216196 |  |  |  |  |
| Corpus callosum | -20,36,10 | -1.915 | 0.027746379 |  |  |  |  |
| Corpus callosum | -4,24,12 | -1.844 | 0.032610297 |  |  |  |  |
| Left ATP | -22,36,4 | -1.767 | 0.038579226 |  |  |  |  |
| Corpus callosum | -10,30,8 | -1.764 | 0.038893938 |  |  |  |  |
| Corpus callosum | -8,12,22 | -1.701 | 0.044449389 |  |  |  |  |
| 2. Corpus callosum | **2,18,16** | **-1.717** | **0.042944014** | **4** | **17.721** | **-0.093** | **0.911** |
| 3. Corpus callosum | **-20,32,16** | **-1.798** | **0.036105275** | **2** | **0.000** | **-0.086** | **0.927** |

Left ATP: Left anterior thalamic projections

Table9:**MDD+ANX+PTSD: VBA**

| Region | MNI | SDM-Z | P value | voxels | I^2^ | Effect size | Metabias test p |
| --- | --- | --- | --- | --- | --- | --- | --- |
| 1. Left striatum Left IN. IFOF  Left ATP  Corpus callosum | **-24,26,10** | **-1.584** | **0.046566894** | **29** | **0.020** | **-0.096** | **0.549** |
| Left striatum | -26,26,14 | -1.397 | 0.051265450 |  |  |  |  |
| Corpus callosum | -20,24,18 | -1.362 | 0.056618841 |  |  |  |  |

Left IN. IFOF: Left inferior network, inferior fronto-occipital fasciculus; Left ATP: Left anterior thalamic projections

**Appendix G:** **DTI analysis methods and statistical characteristics of the participants**

Table10, A total of 44 MDD studies were included

| Study | Quality | P-threshold | Corrected (Uncorrected) | Diffusion directions | Coordinate |
| --- | --- | --- | --- | --- | --- |
|  |  |  |  |  |  |
| Arnold et al_2012 | 10.5 | p < 0.05 | Corrected (FWE) | 30 | MNI |
| Chen et al_2022 | 12 | p < 0.05 | Corrected (FWE) | 99 | MNI |
| Chhetry et al_2016 | 11 | p < 0.001 | Corrected (FWE) | 25 | MNI |
| De Diego et al_2014 | 11 | p < 0.05 | Corrected (FWE) | 15 | MNI |
| Dong et al_2020 | 12 | p < 0.001 | Uncorrected | 32 | MNI |
| Hayashi et al_2014 | 10.5 | p < 0.05 | Corrected (FWE) | 25 | MNI |
| Jia et al_2010 | 12 | p < 0.05 | Corrected | 15 | Talairch |
| Jiang et al_2015 | 10.5 | p < 0.05 | Uncorrected | 25 | MNI |
| Jiang et al_2021 | 11.5 | p < 0.001 | Corrected (FWE) | 32 | MNI |
| Lai et al_2014 | 11 | p < 0.05 | Corrected (FWE) | 30 | MNI |
| Liu et al_2008 | 11 | p < 0.001 | Corrected (FWE) | 25 | Talairch |
| Liu et al_2010 | 10.5 | p < 0.01 | Uncorrected | 12 | MNI |
| Liu et al_2016 | 10.5 | p < 0.05 | Corrected (FWE) | 25 | MNI |
| Lyon et al_2019 | 11 | p < 0.05 | Corrected (FWE) | 42 | MNI |
| Ma et al_2007 | 10.5 | p < 0.001 | Uncorrected | 13 | Talairch |
| Metin et al_2020 | 10.5 | p < 0.05 | Corrected | 16 | MNI |
| Olvet et al_2014 | 11.5 | p < 0.05 | Corrected (FWE) | 25 | MNI |
| Osoba et al_2013 | 12 | p < 0.001 | Corrected (AlphaSim) | 12 | MNI |
| Repple et al_2017 | 10.5 | p < 0.001 | Corrected (AlphaSim) | 20 | MNI |
| Sara et al_2018 | 11.5 | p < 0.05 | Corrected (FWE) | 35 | MNI |
| Seok et al_2013 | 11 | p < 0.01 | Corrected (FWE) | 20 | MNI |
| Srivastava et al_2016 | 10.5 | p < 0.001 | Uncorrected | 65 | MNI |
| Tatham et al_2016 | 11 | p < 0.05 | Corrected (FWE) | 12 | MNI |
| Taylor et al_2015 | 11 | p < 0.05 | Corrected (Monte Carlo) | 20 | MNI |
| Tha et al_2013 | 11 | p < 0.001 | Uncorrected | 12 | MNI |
| Walther et al_2012 | 10.5 | p < 0.05 | Corrected (FDR) | 42 | Talairch |
| Wang et al_2013 | 10.5 | p < 0.01 | Corrected (AlphaSim) | 42 | MNI |
| Wu et al_2011 | 11 | p < 0.001 | Uncorrected | 13 | MNI |
| Xiao et al_2015 | 11 | p < 0.001 | Corrected (CBTM) | 13 | MNI |
| Xu et al_2021 | 11.5 | p < 0.01 | Corrected (FDR) | 25 | MNI |
| Yang et al_2017 | 11 | p < 0.05 | Corrected (TFCE) | 30 | MNI |
| Zheng et al_2021 | 11.5 | p < 0.05 | Corrected (FWE) | 60 | MNI |
| Zhu et al_2011 | 11 | p < 0.05 | Corrected(CBTM） | 13 | MNI |
| Zou et al_2008 | 12 | p < 0.05 | Uncorrected | 15 | Talairch |
| Zuo et al_2012 | 10 | p< 0.005 | Corrected (FDR) | 25 | MNI |
| Guo et al_2023 | 11 | p< 0.05 | Corrected (FWE) | 30 | MNI |
| Vandeloo et al_2023 | 11 | p< 0.05 | Corrected (FWE) | 32 | MNI |
| Winter et al_2022 | 12 | p< 0.05 | Corrected (FEW) | NA | MNI |
| Wu et al_2023  Zhao et al_2024  Zhao et al_2024  Flinken et al_2024  Ma et al_2023 | 10.5  11.5  12  11.5  11.5 | p< 0.05  p<0.05  p<0.05  p<0.05  p<0.05 | Corrected (FEW)  Corrected (Bonferroni)  Corrected (TFCE)  Corrected (TFCE)  Corrected (TFCE) | 32  NA  NA  NA  NA | MNI  MNI  MNI  MNI  MNI |

FEW, family-wise error correction for multiple comparisons; FDR, False Discovery Rate correction for multiple comparisons; thresholding, cluster-based thresholding method; TFCE, threshold-free cluster enhancement; NA

Table11, A total of 9 ANX studies were included

| Study | Quality | P-threshold | Corrected (Uncorrected) | Diffusion directions | Coordinate |
| --- | --- | --- | --- | --- | --- |
|  |  |  |  |  |  |
| Baur et al_2011 | 10.5 | p < 0.05 | Corrected (FWE) | 30 | MNI |
| Kim et al_2014 | 12 | p < 0.05 | Corrected (FWE) | 99 | MNI |
| Kim et al_2013 | 11 | p < 0.001 | Corrected (FWE) | 25 | MNI |
| Lai et al_2013 | 11 | p < 0.05 | Corrected (FWE) | 20 | MNI |
| Lai et al_2016 | 12 | p < 0.05 | Uncorrected | 32 | MNI |
| Lai et al_2013 | 10.5 | p < 0.05 | Corrected (FWE) | 25 | MNI |
| Qiu et al_2014 | 12 | p < 0.05 | Corrected | 15 | MNI |
| Wang et al_2012 | 10.5 | p < 0.05 | Uncorrected | 25 | MNI |
| Zhang et al_2013 | 10.5 | p< 0.05 | Corrected (FEW) | 32 | MNI |

FEW, family-wise error correction for multiple comparisons; FDR, False Discovery Rate correction for multiple comparisons; thresholding, cluster-based thresholding method; TFCE, threshold-free cluster enhancement; NA

| Study | Quality | P-threshold | Corrected (Uncorrected) | Diffusion directions | Coordinate |
| --- | --- | --- | --- | --- | --- |
|  |  |  |  |  |  |
| chen et al 2021 | 10.5 | p < 0.05 | Corrected (FWE) | 30 | MNI |
| odoherty et al 2018 | 12 | p < 0.05 | Corrected (FWE) | 99 | MNI |
| Abe et al 2006 | 11 | p < 0.001 | Corrected (FWE) | 25 | MNI |
| Xi et al 2013 | 11 | p < 0.05 | Corrected (FWE) | 15 | MNI |
| Li et al 2016 | 12 | p < 0.001 | Uncorrected | 32 | MNI |
| Sun et al_2013 | 10.5 | p < 0.05 | Corrected (FWE) | 27 | MNI |
| Zhang et al 2012 | 12 | p < 0.05 | Corrected | 15 | Talairch |
| Fani et al 2012 | 10.5 | p< 0.05 | Corrected (FEW) | Na | MNI |

Table12, A total of 8 PTSD studies were included

**References**

Baur, V., Hänggi, J., & Jäncke, L. (2012). Volumetric associations between uncinate fasciculus, amygdala, and trait anxiety. *BMC Neuroscience*, *13*(1). Scopus. https://doi.org/10.1186/1471-2202-13-4

Gorman, J. M., & Sullivan, G. M. (2000). Neuroanatomical Hypothesis of Panic Disorder, Revised. *Am J Psychiatry*.

Liao, M., Yang, F., Zhang, Y., He, Z., Su, L., & Li, L. (2014). White matter abnormalities in adolescents with generalized anxiety disorder: A diffusion tensor imaging study. *BMC Psychiatry*, *14*(1). Scopus. https://doi.org/10.1186/1471-244X-14-41

Lai, C. H., & Wu, Y. T. (2013). Fronto-occipital fasciculus, corpus callosum and superior longitudinal fasciculus tract alterations of first-episode, medication-naïve and late-onset panic disorder patients. *Journal of Affective Disorders*, *146*(3), 378–382. Scopus. https://doi.org/10.1016/j.jad.2012.09.022

Schmahmann, J. D., & Pandya, D. N. (2007). The Complex History of the Fronto-Occipital Fasciculus. *Journal of the History of the Neurosciences*, *16*(4), 362–377. https://doi.org/10.1080/09647040600620468

Martino, J., Brogna, C., Robles, S. G., Vergani, F., & Duffau, H. (2010). Anatomic dissection of the inferior fronto-occipital fasciculus revisited in the lights of brain stimulation data☆. *Cortex*, *46*(5), 691–699. https://doi.org/10.1016/j.cortex.2009.07.015

Radua, J., Mataix-Cols, D., Phillips, M. L., El-Hage, W., Kronhaus, D. M., Cardoner, N., & Surguladze, S. (2012). A new meta-analytic method for neuroimaging studies that combines reported peak coordinates and statistical parametric maps. *European Psychiatry*, *27*(8), 605–611. https://doi.org/10.1016/j.eurpsy.2011.04.001

Wise, T., Radua, J., Nortje, G., Cleare, A. J., Young, A. H., & Arnone, D. (2016). Voxel-based meta-Analytical evidence of structural disconnectivity in major depression and bipolar disorder. *Biological Psychiatry*, *79*(4), 293–302. Scopus. https://doi.org/10.1016/j.biopsych.2015.03.004
